# The worsening of skeletal muscle atrophy induced by immobilization at the early stage of remobilization correlates with BNIP3-dependent mitophagy

**DOI:** 10.1186/s12891-023-06759-2

**Published:** 2023-08-04

**Authors:** Feng Wang, Ting Zhou, Chen Xu Zhou, Quan Bing Zhang, Hua Wang, Yun Zhou

**Affiliations:** 1 Department of Rehabilitation Medicine, the Second Affiliated Hospital of Anhui Medical University, No.678 Furong Road, Hefei, 230601 China; 2 Research Center for Translational Medicine, the Second Affiliated Hospital of Anhui Medical University, Hefei, 230601 China; 3 Department of Toxicology, School of Public Health, Anhui Medical University, Hefei, 230032 China; 4 Key Laboratory of Environmental Toxicology of Anhui Higher Education Institutes, Hefei, 230032 China

**Keywords:** Muscle atrophy, Remobilization, BNIP3-denpendent mitophagy, ROS, HIF-1α

## Abstract

**Background:**

Recent studies have shown that immobilization enhances reactive oxygen species (ROS) production and mitophagy activity in atrophic skeletal muscle. However, there are relatively few studies examining the biological changes and underlying mechanisms of skeletal muscle during remobilization. In this study, we aimed to investigate the effects of remobilization on skeletal muscle and explore the role of BNIP3-dependent mitophagy in this process.

**Methods:**

Thirty rats were randomly divided into six groups based on immobilization and remobilization time: control (C), immobilization for two weeks (I-2w), and remobilization for one day (R-1d), three days (R-3d), seven days (R-7d), and two weeks (R-2w). At the end of the experimental period, the rectus femoris muscles were removed and weighed, and the measurements were expressed as the ratio of muscle wet weight to body weight (MWW/BW). Sirius Red staining was performed to calculate the values of cross-sectional area (CSA) of rectus femoris. Oxidative fluorescent dihydroethidium was used to evaluate the production of ROS, and the levels of superoxide dismutase (SOD) were also detected. The morphological changes of mitochondria and the formation of mitophagosomes in rectus femoris were examined and evaluated by transmission electron microscope. Immunofluorescence was employed to detect the co-localization of BNIP3 and LC3B, while Western blot analysis was performed to quantify the levels of proteins associated with mitophagy and mitochondrial biogenesis. The total ATP content of the rectus femoris was determined to assess mitochondrial function.

**Results:**

Within the first three days of remobilization, the rats demonstrated decreased MWW/BW, CSA, and ATP concentration, along with increased ROS production and HIF-1α protein levels in the rectus femoris. Results also indicated that remobilization triggered BNIP3-dependent mitophagy, supported by the accumulation of mitophagosomes, the degradation of mitochondrial proteins (including HSP60 and COX IV), the elevation of BNIP3-dependent mitophagy protein markers (including BNIP3, LC3B-II/LC3B-I, and Beclin-1), and the accumulation of puncta representing co-localization of BNIP3 with LC3B. Additionally, PGC-1α, which is involved in the regulation of mitochondrial biogenesis, was upregulated within the first seven days of remobilization to counteract this adverse effect.

**Conclusion:**

Our findings suggested that BNIP3-denpendent mitophagy was sustained activated at the early stages of remobilization, and it might contribute to the worsening of skeletal muscle atrophy.

**Supplementary Information:**

The online version contains supplementary material available at 10.1186/s12891-023-06759-2.

## Introduction

Skeletal muscle atrophy is a prevalent syndrome that results in significant muscle mass loss and reduced muscle function, leading to a decrease in quality of life and increased risk of chronic disease [1]. Immobilization, a common orthopedic treatment used to ease pain and reduce inflammation in patients with musculoskeletal damage, is one of the leading causes of muscle atrophy. Investigating the mechanisms responsible for muscle recovery during immobilization is of clinical relevance, as it better recapitulates events involved in fractures and bed rest-associated immobilization compared to unloading models [2]. In our previous rabbit model of extending knee joint contracture, as a critical component of myogenic contracture, skeletal muscle atrophy occurred during immobilization and recovered gradually during remobilization. Interestingly, our previous report suggested that the protein level of MyoD was further decreased during the early phase of remobilization with sustained activation of the NF-κB/HIF-1α pathway [3]. MyoD is an integral component of ubiquitin-proteasome-dependent proteolysis pathway, as it is one of the myogenic regulatory factors that plays a crucial role in inhibiting muscle proteolysis [3]. Although previous research has demonstrated that sustained activation of the ubiquitin-proteasome system and/or apoptosis during early remobilization worsened skeletal muscle atrophy, the underlying mechanisms remain incompletely understood [2].

The loss of muscle mass resulting from immobilization is primarily driven by alterations in muscle protein turnover. This wasting has been extensively investigated and occurs due to an imbalance between protein synthesis and breakdown rates, as well as cell death and regeneration processes [4]. According to previous research, skeletal muscle protein turnover is primarily regulated by the activation of the ubiquitin-proteasome system [5–7]. However, recent studies have suggested that protein degradation mediated by the autophagy-lysosomal pathway may also play a crucial role in muscle atrophy resulting from immobilization [8, 9].

**Fig. 1:**
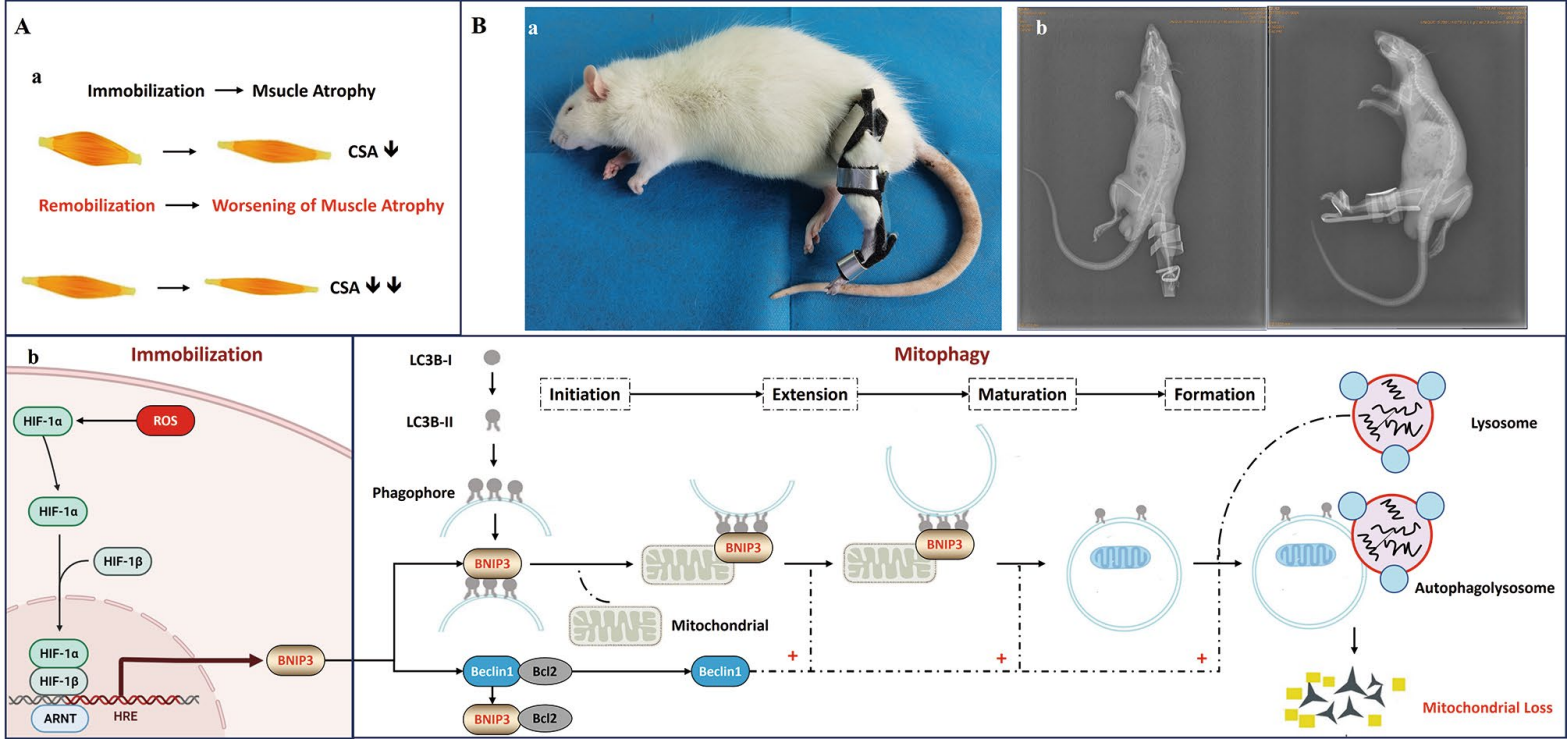
(**A**) Research hypothesis. (**a**) Skeletal muscle atrophy induced by immobilization worsened at the early stage of remobilization. This phenomenon may be related to hyperactive mitophagy. (**b**) The molecular mechanism of BNIP3-mediated mitophagy. Under induction by ROS, BNIP3 can directly bind with LC3B-II and mediate the interaction of phagophores, impairing mitochondrial function. In addition, BNIP3 competes the binding site of Bcl-2 protein with Beclin-1 and releases Beclin-1. The free Beclin-1 subsequently forms the complexes and participates in autophagosome extension, maturation, and formation. Finally, the mitochondria are surrounded by autophagosomes and subsequently broken up by lysosomes. (**B**) Animal model. (**a**) Unilateral immobilization of the rat knee joint at full extension using a shaped aluminum splint. (**b**) X-ray film was applied to confirm the left knee joint of rat was fixed at full extension

Normal mitochondrial function is crucial in maintaining skeletal muscle energy metabolism [10]. Mitophagy, a process involving the removal of fragmented mitochondria, plays an essential role in mitochondrial quality control [11]. Temperately activated mitophagy contributes to remove damaged or superfluous mitochondria, and thereby maintaining mitochondrial function and adapting to stress [12]. However, overactivated mitophagy causes excessive mitochondrial loss, to result in cellular bioenergy deficit and cell death [13]. The novel mitophagy receptor, Bcl2/adenovirus E1B 19 kDa protein-interacting protein 3 (BNIP3), has been shown to be expressed in skeletal muscle [14–16]. Multiple studies have demonstrated that reactive oxygen species (ROS) increase the expression of hypoxia-inducible factor (HIF)-1α and its downstream targets, such as BNIP3. Subsequently, BNIP3 interacts with microtubule-associated protein 1 light chain 3 (LC3) to form a mitochondria-BNIP3-LC3-autophagosome complex, commencing the process of mitophagy [17–19]. Previous studies in animals and humans have shown that immobilization increases the expression of BNIP3 and activates the process of mitophagy in skeletal muscle [14–16]. Importantly, previous research suggests that age-related skeletal muscle atrophy arises, in part, from the release of ROS and the hyperactive degradation of mitochondria through mitophagy, which can lead to a reduction in the number and size of myofibers [20]. However, there are few studies that have been conducted on the BNIP3-dependent mitophagy in skeletal muscle atrophy during remobilization. Based on previous research, we hypothesize that hyperactive BNIP3-dependent mitophagy may contribute to the progression of muscle atrophy at the early stage of remobilization. (Fig. 1. A).

Our team has conducted previous studies in vivo using rabbits and confirmed that sustained expression of HIF-1α and hyperactive autophagy play a crucial role in skeletal muscle atrophy during immobilization [3, 21]. In this current study, we aim to investigate the role of BNIP3-dependent mitophagy at the early stage of remobilization. To this end, we have developed a novel rat model involving external extending knee joint immobilization to explore the underlying mechanisms.

## Methods

### Animals

The Institutional Animal Care and Use Committee of Anhui Medical University approved all experimental procedures, including animal use and care protocols (LLSC20221126). A total of thirty adult male Sprague-Dawley rats (approximately 3 months old, 200-250 g) were provided from animal laboratory center of Anhui Medical University (Hefei, China) and randomized to 6 groups: group C (control group), group I-2w (2w-immobilization), group R-1d (1d-remobilization after 2w-immobilization), group R-3d (3d-remobilization after 2w-immobilization), group R-7d (7d-remobilization after 2w-immobilization), group R-2w (2w-remobilization after 2w-immobilization), with 5 rats in each group. All rats were kept in the same environment, with one rat per cage, at a temperature of 22-24 °C, under a 12-hr light/dark cycle, and were given free access to food and water. During the experimental period, the rats in each group had freedom of movement within the cage, except for the immobilized part.

### Model creation

The surgical procedures were performed under general anesthesia, with an intraperitoneal injection of 2% pentobarbital sodium salt solution (40 mg/kg). First, aluminum splint was cut into a special shape from a whole aluminum plate and was molded into another shape that fitted the left lower limb of rat. Then absorbent cotton and aluminum splint were tightly bonded by instant adhesive. Rats were placed on a surgical table in the supine position, and the shaped aluminum splint was applied to immobilize the left knee joint (Fig. 1. B. a). X-ray film was applied to confirm the left knee joint of rat was fixed at full extension (Fig. 1. B. b).

### Skeletal muscle tissue preparation

**Fig. 2:**
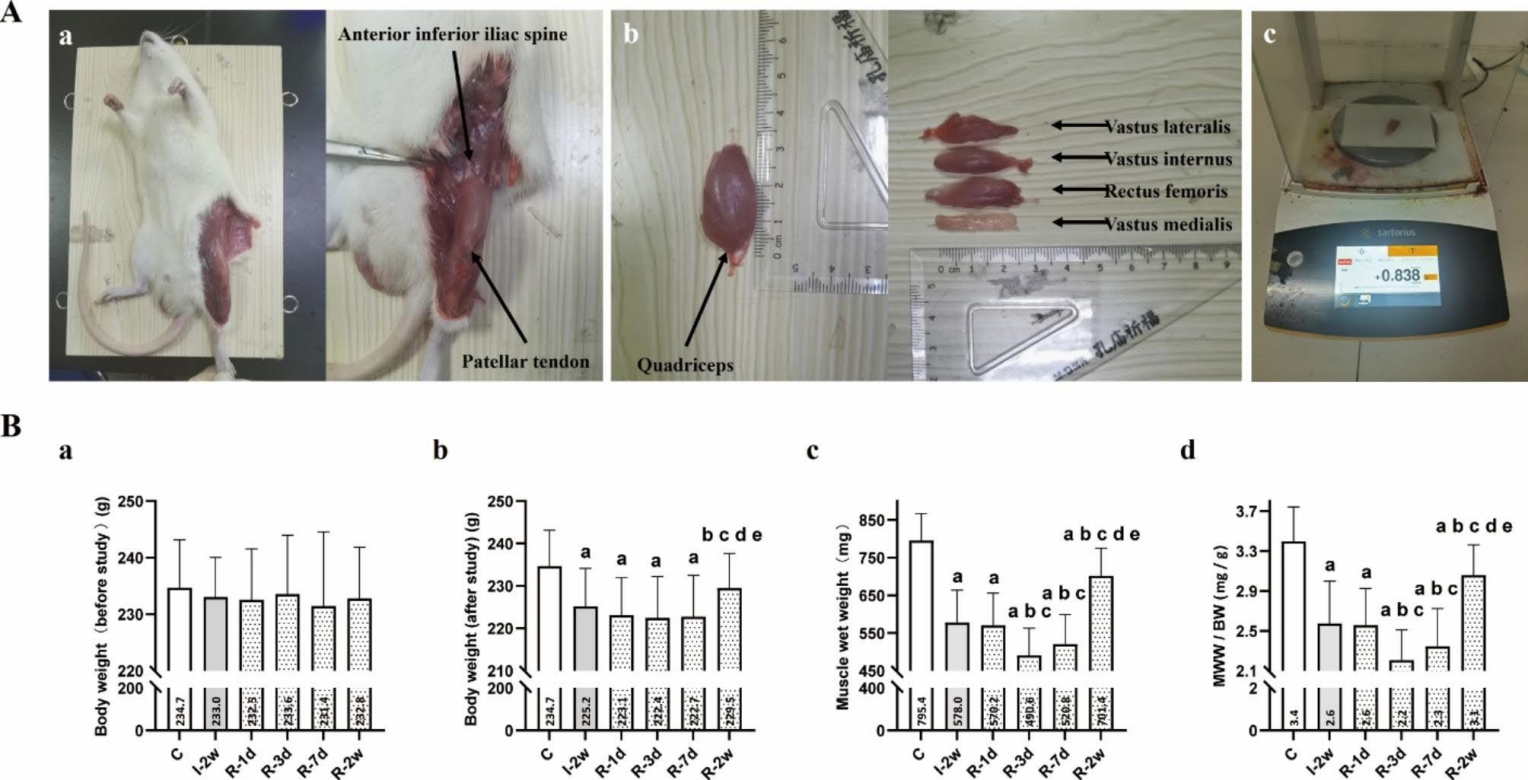
(**A**) Process of rectus femoris tissue preparation. (**a**) The skin was resected from the fascia by blunt dissection to properly view quadriceps, anterior inferior iliac spine, and patellar tendon. (**b**) Rectus femoris were completely separated from quadriceps. (**c**) The wet weight of the rectus femoris was obtained using an electronic analytical balance. (**B**) The value of body weight (before study) (**g**), body weight (after study) (**g**), muscle wet weight of rectus femoris (mg), and MWW/BW (mg/g) in each group (n = 5 per group). ^a^*P* < 0.05 vs. group C, ^b^*P* < 0.05 vs. group I-2w, ^c^*P* < 0.05 vs. group R-1d, ^d^*P* < 0.05 vs. group R-3d, ^e^*P* < 0.05 vs. group R-7d

To examine the effects of immobilization and remobilization on skeletal muscle, we selected the rectus femoris as our model. In previous studies, we found that the level of atrophy was severe in the rectus femoris, which was fixed in a shortened position during knee extension immobilization [7]. Upon completion of the experimental period, rats were euthanized with an intraperitoneal injection of 120 mg/kg of pentobarbital sodium salt. The rectus femoris muscles were removed, trimmed of excess tissue, and weighed (Fig. 2. A). Muscle wet weight (MWW, mg) of the rectus femoris was measured using our laboratory scale [21], expressed in absolute terms, and as the ratio of muscle wet weight to body weight (MWW/BW, mg/g). Four muscle tissue samples, each measuring 0.5 cm × 0.5 cm × 0.5 cm, were collected from the middle of the rectus femoris. Of these samples, two were used for sirius red staining and ROS production measurement, while the others were immediately frozen in isopentane chilled with liquid nitrogen and stored at -80 °C until subsequent analysis.

### Sirius red staining

The rectus femoris tissues were fixed with 4% paraformaldehyde and then embedded in paraffin. Serial transverse 10-µm sections were cut using a cryostat from the middle part of the muscle belly for histological analysis. Sirius Red staining was carried out according to the instructions of the Sirius Red Stain Kit (Sbjbio, BP-DL027, China). According to our previous research, the cross-sectional area (CSA) of individual myofibers and the percentage of collagen relative area was photographed using a TE2000-U inverted microscope under a 10 × 40 magnification (Nikon Corporation, Japan) and measured using Image J software (National Institutes of Health, USA) [3, 22]. To ensure the CSA was calculated from at least 200 myofibers per individual, six randomly selected fields of view were analyzed in each section. The acquisition of images was performed by the same experimenter, and he was blinded to groups during image acquisition and data analysis.

### Detection of ROS

According to previous research, the measurement of ROS production was evaluated using oxidative fluorescent dihydroethidium (DHE) [23]. DHE is a cell-permeable agent and it interacts with nucleic acids. DHE produces red fluorescence when oxidized to ethidium bromide by ROS, including superoxide anion. We detected the red light by a fluorescence microscope (Olympus, Japan) with a rhodamine filter (excitation 490 nm, emission 590 nm). The thin frozen sections were incubated with 0.1% DHE (Beyotime, S0063, China) solution for 30 min at 37 ℃ in a dark box, rinsed with PBS, and viewed using a fluorescence microscope under a 400× magnification field (filter with excitation at 545 nm). Densitometric analysis of DHE fluorescence was performed using the Image J software (National Institutes of Health, USA) in randomly selected six images per section and reported as a percentage of group C. The acquisition of images was performed by the same experimenter, and he was blinded to groups during image acquisition and data analysis.

An over-production of ROS can take away numerous ROS-scavenging enzymes, such as superoxide dismutase (SOD). Therefore, SOD is commonly used for indirectly evaluating the level of ROS. As our previous study [24], the samples of rectus femoris were added with nine times the volume of normal saline with the proportion of weight (g): volume (ml) = 1:9 to prepare the 10% tissue homogenate at 2500 rpm for 10 min using a commercial kit (Nanjing Jiancheng Bioengineering Institute, A001-3, China). The activity of SOD in the tissue homogenate were determined according to the manufacturer’s protocol.

### Transmission electron microscope imaging

The muscle blocks were prepared and soaked immediately in 2.5% glutaraldehyde. After 6–8 h at 4 °C, they were cut into 1 mm thick coronal slices. Next, the samples were rinsed with PBS (0.1 M) before being postfixed by osmium tetroxide for 1–2 h. The muscle coronal slices were dehydrated through a graded series of alcohol and acetone. Subsequently, we used epoxy resin for embedding prior to slicing of the ultra-thin sections. Then, double staining by uranyl acetate and lead citrate was performed. Finally, the images were acquired by a JEM-1400 transmission electron microscope (JEOL Ltd, Japan).

### Immunofluorescence

Thin sections of rectus femoris were fixed for 1 h with 4% paraformaldehyde. Nonspecific binding sites in the slides were blocked using 10% normal goat serum. Subsequently, the slides were incubated with the mixture containing primary antibodies against LC3B (ABclonal, A17424, China; dilution 1:100) and BNIP3 (ABclonal, A5683, China; dilution 1:100) at 37 ℃ for 2 h. After being washed with PBS, the sections were incubated using the mixture containing secondary antibodies of FITC Goat Anti-rabbit IgG (H + L) (ABclonal, AS011, China; dilution 1:100) and Cy3 Goat Anti-mouse IgG (H + L) (ABclonal, AS008, China; dilution 1:100) at 37 ℃ for 1 h. The sections were stained with DAPI (Beyotime, C1005, China) in room temperature for 5 min. Each section was observed by a fluorescence microscope (Olympus, Japan) under a 400× magnification field. For co-localization of LC3B and BNIP3 (yellow puncta) analysis, six randomly selected fields of view were analyzed in each section by the Image J software (National Institutes of Health, USA). The acquisition of images was performed by the same experimenter, and he was blinded to groups during image acquisition and data analysis. According to our previous study, the mean number of yellow fluorescence points in the individual muscle fibers in each visual field was calculated for statistical analysis [21]. Additionally, the number of muscle cell containing yellow fluorescence points per nucleus (DAPI stained) was counted and calculated as a percentage.

### Western blot analysis

To obtain total proteins, the samples of rectus femoris were ground into powder with liquid nitrogen using a grinder and homogenized in RIPA buffer (Beyotime, P0013B, China) containing protease and phosphatase inhibitor cocktail at 4℃. Homogenates were centrifuged at 12,000 × g for 30 min three times at 4℃, and the resulting supernatants were collected. To obtain cytosolic and nuclear proteins, a Nuclear and Cytoplasmic Protein Extraction Kit (Beyotime, P0027, China) was used as per manufacturer’s instruction. The protein concentrations were determined using BCA (Bicinchoninic acid) Protein Quantitative Kit (Beyotime, P0012, China). Protein lysates were separated on a 10% sodium dodecyl sulfate-polyacrylamide electrophoresis gel and transferred on to polyvinylidene fluoride membranes (Millipore, USA). After being blocked with five-percent non-fat dry milk in Tris-buffered saline Tween-20 (TBST) at room temperature for two hours, the membranes were incubated with rabbit anti-HIF-1α (Abcam, ab179483, China; dilution 1:1000), rabbit anti-PGC-1α (ABclonal, A12348, China; dilution 1:1000), rabbit anti-HSP60 (Abcam, ab190828, China; dilution 1:1000), rabbit anti-COX IV (Abcam, ab202554, China; dilution 1:2000), rabbit anti-PINK1 (Abcam, ab300623, China; dilution 1:1000), rabbit anti-Parkin (Abcam, ab73015, China; dilution 1:1000), rabbit anti-Beclin-1 (Abcam, ab207612, China; dilution 1:1000), rabbit anti-BNIP3 (Abcam, ab109362, China; dilution 1:1000), rabbit anti-LC3B (Abcam, ab63817, China; dilution 1:2000) at four degrees Celsius overnight. On the second day, after being washed in TBST solution three times for 10 min per wash, the membranes were incubated with peroxidase-conjugated affinipure goat anti-rabbit IgG-HRP (Abcam, ab6721, China; dilution 1:5000) as the secondary antibody for two hours at room temperature. After being washed three times with TBST or 10 min per wash, the membranes were evaluated with the enhanced chemiluminescence system in accordance with the manufacturer’s instructions. The band densities were quantified using Image J software (National Institutes of Health, USA). The relative protein levels were calculated by comparison with the amount of GAPDH (Abcam, ab9485, China; dilution 1:1000) or Histone H3 (Abcam, ab1791, China; dilution 1:2000) as a loading control.

### ATP assessment

The total ATP content was examined by the ATP Assay Kit (Beyotime, China, S0026). As described in the instruction, the samples of rectus femoris were lysed in lysis buffer with centrifugation at 4 °C and 12,000 ×g for 5 min. Then, the supernatant was extracted for the ATP assay. Before the assay, the ATP working reagents were added into a micro-well for 5 min at 37 °C. The supernatant (20 µl) was added, then the values of relative light unit (RLU) were obtained by a modular multitechnology microplate reader (Thermo Fisher Scientific, Massachusetts, USA). The ATP concentrations were calculated through the standard curve method. Then the protein levels of different samples were acquired using a BCA Protein Quantitative Kit (Beyotime, P0012, China). Ultimately, the ATP levels were displayed in the form of nanomoles per milligrams.

### Statistical analysis

All the results were expressed as mean ± SD for each group. The assumptions of normality of data and homogeneity of variances between the groups were analyzed by SPSS 26.0 (IBM Corp, Armonk, NY, USA). Comparisons between multiple-group means were performed using by one-way analysis of variance (ANOVA) with Tukey’s as post hoc test when the data showed a normal distribution. All statistical graphs were processed using GraphPad Prism 9 (GraphPad Scientific, San Diego, CA, USA).

## Results

### Model assessment

**Fig. 3:**
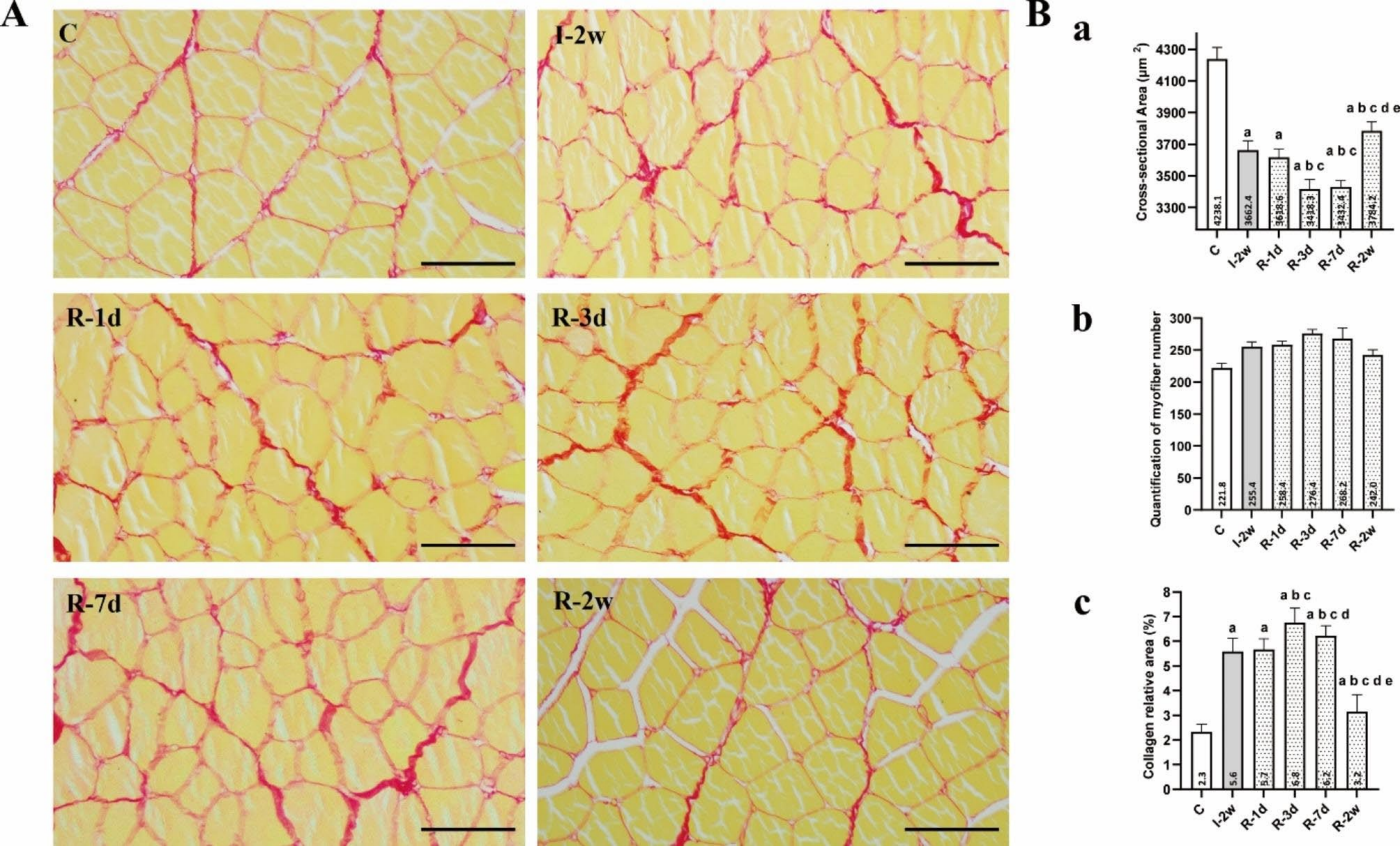
Rectus femoris atrophy induced by immobilization worsened within the first three days of remobilization. (**A**) Representative Sirius red staining images in each group (n = 5 per group), scale bar = 200 μm. (**B**) (**a**) The value of cross-sectional area of the rectus femoris (µm^2^). ^a^*P* < 0.05 vs. group C, ^b^*P* < 0.05 vs. group I-2w, ^c^*P* < 0.05 vs. group R-1d, ^d^*P* < 0.05 vs. group R-3d, ^e^*P* < 0.05 vs. group R-7d. (**b**) Quantification of myofiber number in each group (n = 5 per group). (**c**) The percentage of collagen relative area (%). ^a^*P* < 0.05 vs. group C, ^b^*P* < 0.05 vs. group I-2w, ^c^*P* < 0.05 vs. group R-1d, ^d^*P* < 0.05 vs. group R-3d, ^e^*P* < 0.05 vs. group R-7d

During the immobilization and remobilization period, all rats survived. The rats with immobilized left knees could move freely within the cage and also eat and drink by themselves. No rat developed prolonged edema or acute inflammation. The MWW/BW and cross-sectional area were significantly lower in group I-2w than in the group C (*P* < 0.05, Fig. 2. B & Fig. 3). Serious skeletal muscle atrophy was developed after two weeks of immobilization. Apart from this, the percentage of collagen relative area was significantly higher in group I-2w than in the group C (*P* < 0.05, Fig. 3). Consequently, myofibrosis was also observed.

### Body and muscle weight

The results of the body and muscle wet weights were listed in Fig. 2. B. There were no significant differences in body weight among the six groups of rats by before the experiment (*P* > 0.05). Both MWW and MWW/BW were significantly lower in group R-3d compared to group I-2w (*P* < 0.05). No significant difference in both MWW and MWW/BW between group R-3d and group R-7d was seen (*P* > 0.05). With the development of remobilization, both MWW and MWW/BW were increased in group R-2w by compared with group R-7d (*P* < 0.05).

### Muscle fiber cross-sectional area

To examine the effects of remobilization, we performed Sirius red staining and calculated the CSA values of rectus femoris. As presented in Fig. 3, The results showed that an obviously increasing trend of the CSA values occurred after the first three days of remobilization. However, the results showed that a decreasing trend of the CSA values was visible within the first three days of remobilization. Compared with the group I-2w, the group R-3d had the most obvious reduction in the CSA value (*P* < 0.05). Altogether, these data suggest a pronounced reduction of rectus femoris volume at the early stage of remobilization, in other words, a worsening of skeletal muscle atrophy.

### The level of ROS and HIF-1α

**Fig. 4:**
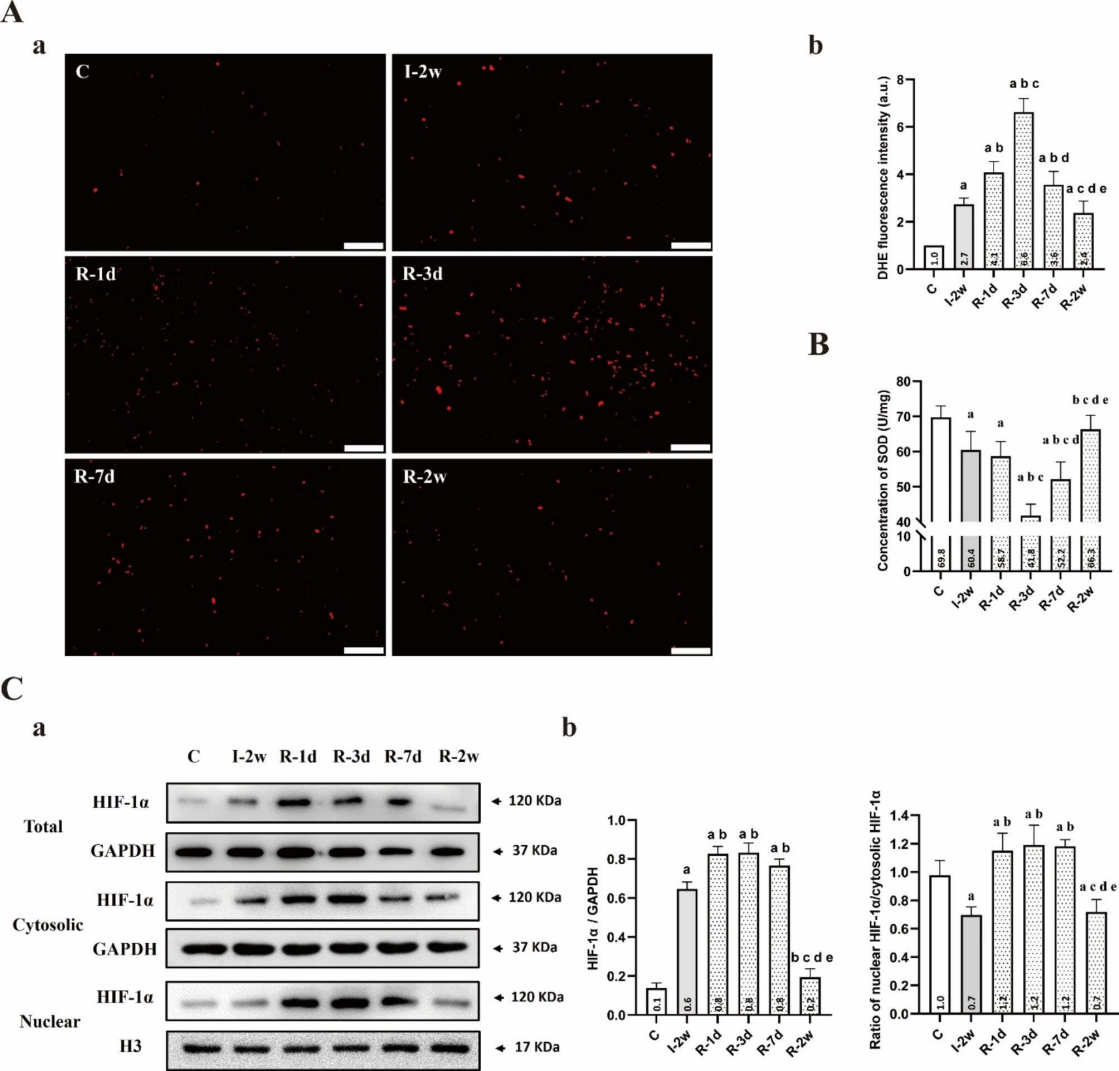
Remobilization enhanced ROS production and HIF-1α expression in the rectus femoris within the first three days. (**A**) (**a**) Representative DHE staining images to measure ROS production of the rectus femoris in each group (n = 5 per group). Scale bar = 50 μm. (**b**) DHE fluorescence intensity (as a percentage of group C). ^a^*P* < 0.05 vs. group C, ^b^*P* < 0.05 vs. group I-2w, ^c^*P* < 0.05 vs. group R-1d, ^d^*P* < 0.05 vs. group R-3d, ^e^*P* < 0.05 vs. group R-7d. (**B**) Concentration of SOD (U/mg) in each group (n = 5 per group). ^a^*P* < 0.05 vs. group C, ^b^*P* < 0.05 vs. group I-2w, ^c^*P* < 0.05 vs. group R-1d, ^d^*P* < 0.05 vs. group R-3d, ^e^*P* < 0.05 vs. group R-7d. (**C**) (**a**) The total HIF-1α/GAPDH, cytosolic HIF-1α/GAPDH, and nuclear HIF-1α/ Histone H3 of the rectus femoris in each group (n = 3 per group). (**b**) The average protein level and the ratio of nuclear HIF-1α/cytosolic HIF-1α for. ^a^*P* < 0.05 vs. group C, ^b^*P* < 0.05 vs. group I-2w, ^c^*P* < 0.05 vs. group R-1d, ^d^*P* < 0.05 vs. group R-3d, ^e^*P* < 0.05 vs. group R-7d. All the gels were trimmed and the samples derive from the same experiment and that gels/blots were processed in parallel

ROS production in rectus femoris was measured by DHE stain as DHE fluorescence intensity. As presented in Fig. 4. A, DHE fluorescence intensity was significantly greater in group I-2w compared with group C (*P* < 0.05). Moreover, DHE fluorescence intensity was increased by remobilization within the first three days of remobilization. Compared with the group I-2w, the group R-3d had the most obvious growth (*P* < 0.05). A subsequent decrease was observed after the first three days of remobilization, and compared with the group R-3d, the group R-2w had the most obvious reduction (*P* < 0.05). Additionally, a negative correlation was observed between the activity of SOD and the DHE fluorescence intensity, with the most significant reduction observed in the activity of SOD of group R-3d compared to group I-2w (*P* < 0.05, Fig. 4. B).

The protein level for total HIF-1α were significantly greater in group I-2w compared with group C (*P* < 0.05, Fig. 4. C). As presented in Fig. 4. C, the results showed that a stable high level of the protein level for total HIF-1α was visible within the first seven days of remobilization. Compared with the group I-2w, the group R-3d had the most obvious growth (*P* < 0.05). Increased ratios of nuclear HIF-1α to cytosolic HIF-1α were evident in group R-1d, R-3d and R-7d relative to group I-2w. Similar results to those observed at the CSA values have been identified at the protein level for total HIF-1α. A subsequent decrease was observed after the first seven days of remobilization, and compared with the group R-7d, the group R-2w had the most obvious reduction (*P* < 0.05).

### BNIP3-dependent mitophagy

**Fig. 5:**
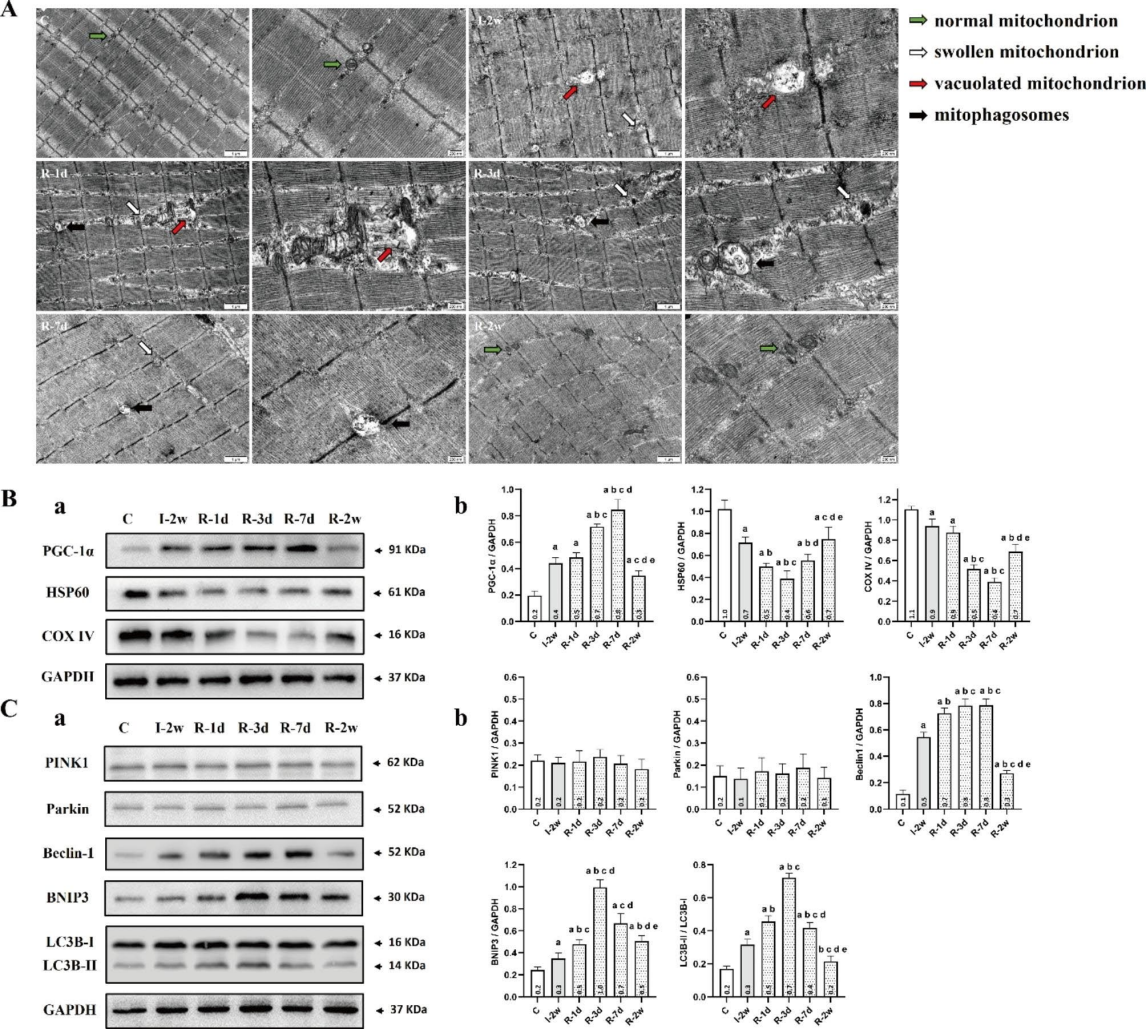
Remobilization increased the activation of mitophagy in rectus femoris within the first three days. (**A**) Mitochondrial biosynthesis was upregulated during remobilization to counteract this adverse effect. Mitochondria were generally normal in morphology and number in group C. A large amount vacuolated mitochondria and noted loss of mitochondria number were detected in group I-2w. Accumulation of mitophagosomes was observed in group R-1d and R-3d. (**B**) (**a**) The PGC-1α/GAPDH, HSP60/GAPDH, COX IV/GAPDH of the rectus femoris in each group (n = 3 per group). (**b**) The average protein level for. ^a^*P* < 0.05 vs. group C, ^b^*P* < 0.05 vs. group I-2w, ^c^*P* < 0.05 vs. group R-1d, ^d^*P* < 0.05 vs. group R-3d, ^e^*P* < 0.05 vs. group R-7d. (**C**) (**a**) The PINK1/GAPDH, Parkin/GAPDH, Beclin-1/GAPDH, BNIP3/GAPDH, LC3B-II/LC3B-I of the rectus femoris in each group. (n = 3 per group). (**b**) The average protein level for. ^a^*P* < 0.05 vs. group C, ^b^*P* < 0.05 vs. group I-2w, ^c^*P* < 0.05 vs. group R-1d, ^d^*P* < 0.05 vs. group R-3d, ^e^*P* < 0.05 vs. group R-7d. All the gels were trimmed and the samples derive from the same experiment and that gels/blots were processed in parallel

The morphological changes of mitochondria and the formation of mitophagosomes in rectus femoris were examined and evaluated by transmission electron microscope (TEM). Mitochondria were generally normal in morphology and number in group C. A large amount vacuolated mitochondria and noted loss of mitochondria number were detected in group I-2w. Importantly, accumulation of mitophagosomes was observed within the first three days of remobilization (Fig. 5. A).

The average protein level for BNIP3 was significantly greater in group I-2w compared with group C (*P* < 0.05, Fig. 5. C). As presented in Fig. 5. C, the results showed that an increasing trend of the protein level for BNIP3 was visible within the first three days of remobilization. A subsequent decrease was observed after the first three days of remobilization, and compared with the group R-3d, the group R-2w had the most obvious reduction (*P* < 0.05, Fig. 5. C).

**Fig. 6:**
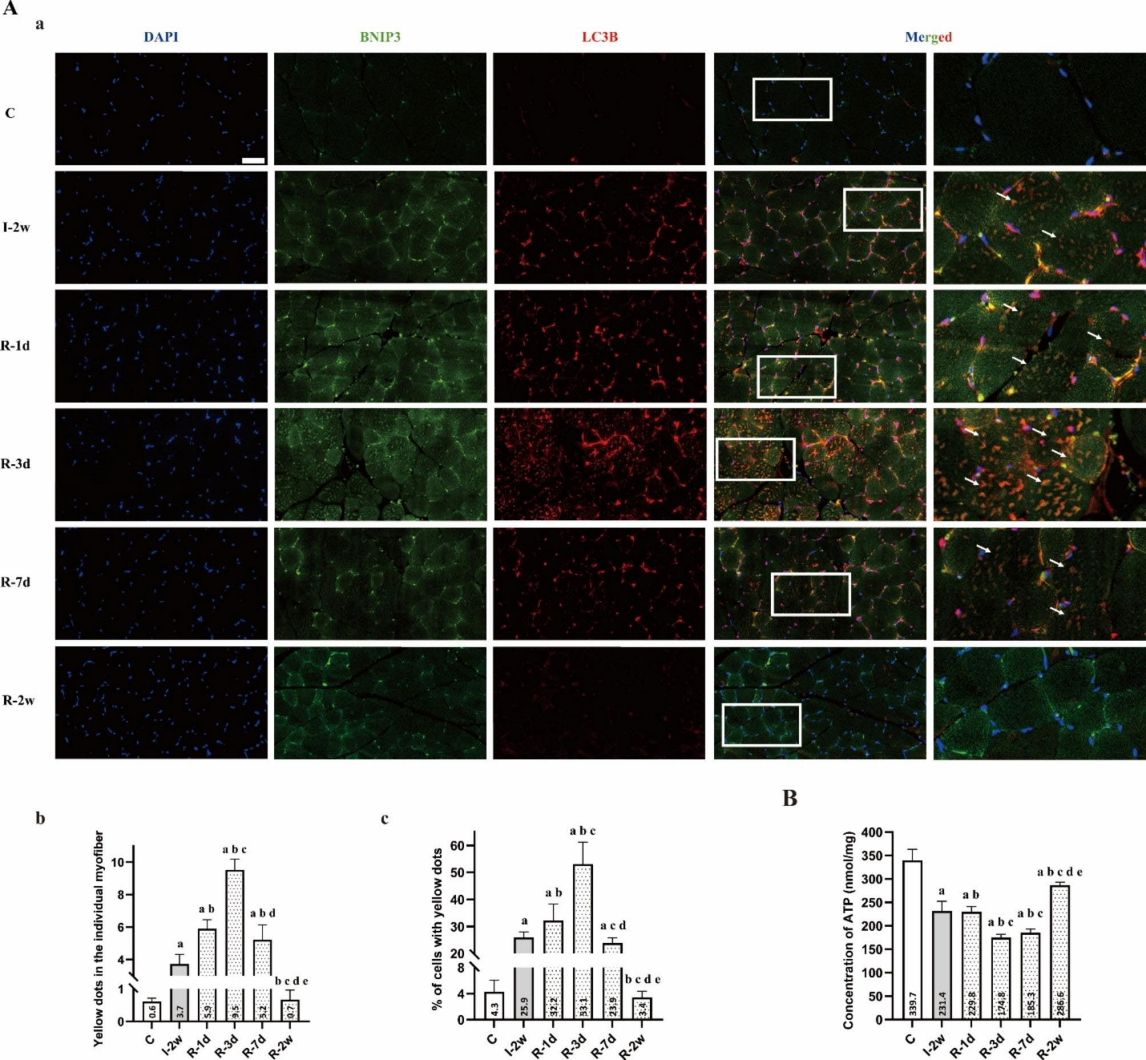
(**A**) Remobilization increased the puncta of co-localization of LC3B and BNIP3 in rectus femoris within the first three days. (**a**) Immunofluorescence assay (n = 5 per group) was used to localize the expression of LC3B (red) and BNIP3 (green). Nuclei were marked with DAPI (blue). Scale bar = 50 μm. (**b**) Quantitative analysis of yellow dots in the individual myofiber. ^a^*P* < 0.05 vs. group C, ^b^*P* < 0.05 vs. group I-2w, ^c^*P* < 0.05 vs. group R-1d, ^d^*P* < 0.05 vs. group R-3d. (**c**) Quantitative analysis of percentage of cells with yellow dots. ^a^*P* < 0.05 vs. group C, ^b^*P* < 0.05 vs. group I-2w, ^c^*P* < 0.05 vs. group R-1d, ^d^*P* < 0.05 vs. group R-3d. (**B**) Concentration of ATP (nmol/mg) in each group (n = 5 per group). ^a^*P* < 0.05 vs. group C, ^b^*P* < 0.05 vs. group I-2w, ^c^*P* < 0.05 vs. group R-1d, ^d^*P* < 0.05 vs. group R-3d, ^e^*P* < 0.05 vs. group R-7d

To further characterize the observed mitophagy, the level of mitochondrial protein and autophagy-associated protein were assessed. As shown in Fig. 5. B & C, the protein level for LC3B-II and Beclin-1 in group I-2w were higher than those of group C (*P* < 0.05), and the protein level for HSP60 and COX IV in group I-2w were lower than group C (*P* < 0.05), which demonstrated that immobilization might activate mitophagy. As presented in Fig. 5. C, an increasing trend of the protein level for LC3B-II were visible within the first three days of remobilization. Apart from this, the results also showed that a decreasing trend of the protein level for HSP60 and COX IV. Compared with the group I-2w, the group R-3d had the most obvious reduction in the protein level for HSP60 (*P* < 0.05, Fig. 5. B), and the group R-7d had the most obvious reduction in the protein level for COX IV (*P* < 0.05, Fig. 5. B). Interestingly, as shown in Fig. 6. B, the increase in the protein level for Beclin-1 was persistent for seven days. However, as presented in Fig. 5. C, the protein level for PINK1 and Parkin remained stable at low levels in all groups. These results might indicate that overactivated BNIP3- dependent mitophagy contributed to the worsening of skeletal muscle atrophy induced by immobilization at the early stage of remobilization.

In order to prove this conjecture, we examined the co-localization of green fluorescence-labelled BNIP3 and red fluorescence-labelled LC3B. As presented in Fig. 6. A, we found that there was a significant increment of the mean number of the co-localization of LC3B and BNIP3 (yellow fluorescence points) in the individual muscle fibers, in group I-2w compared with the group C. Importantly, the results showed that the mean number of the co-localization of LC3B and BNIP3 in the individual muscle fibers in group R-3d were more than those in group I-2w (*P* < 0.05). As above, the results of immunofluorescence staining with sections of rectus femoris provided further evidence for BNIP3-mediated mitophagy in atrophic skeletal muscle at the early stage of remobilization.

### Total ATP content

As illustrated in Fig. 6. B, our results demonstrated a decreasing trend in the total ATP content. Specifically, the group undergoing remobilization for 7 days (R-7d) exhibited the most significant reduction in total ATP content compared to the group receiving immobilization for 2 weeks (I-2w) (*P* < 0.05). Subsequently, we observed an increase in the total ATP content after the first seven days of remobilization. In addition, the group undergoing remobilization for 2 weeks (R-2w) displayed the most significant increase in total ATP content compared to the group R-7d (*P* < 0.05).

### Mitochondrial biogenesis

As presented in Fig. 5. B, the results also showed that an increasing trend of the protein level for PGC-1α was visible within the first seven days of remobilization. Correspondingly, the protein level for HSP60 and COX IV in group R-2w was higher than group R-3d (*P* < 0.05).

## Discussion

As the population ages and the transportation and construction industries rapidly develop, the number of patients with limb immobilization due to aging and trauma continues to increase annually. To maintain an optimal quality of life, preventing skeletal muscle atrophy after limb immobilization is critical. Thus, it is necessary to clarify the mechanisms that regulate skeletal muscle atrophy during immobilization and remobilization. The involvement of mitophagy in skeletal muscle has been increasingly recognized over the past decade. Previous research has been demonstrated that mitophagy activity and mitochondrial ROS levels are increased during disuse-induced muscle atrophy [15]. Despite these findings, only a limited number of studies have focused on the recovery of skeletal muscle during remobilization, and the mechanism is not yet fully understood. In the present study, we aimed to investigate the involvement of BNIP3-dependent mitophagy in muscle atrophy with prolongation of remobilization time.

The use of a novel aluminum splint immobilization in this experiment induced considerable atrophy and fibrosis of the rectus femoris, which is consistent with previous findings from casted-immobilization models in our previous studies [3, 7]. The observed atrophy and fibrosis were attributed to a marked decrease in the amount of activity induced by the aluminum splint immobilization used. Taken together, these findings suggest that the novel immobilization model used in this study is appropriate and reliable for modeling skeletal muscle atrophy.

Our present study reveals a decreasing trend in CSA values and MWW/BW of the rectus femoris within the first three days of remobilization, consistent with previous findings suggesting the involvement of mechanisms exacerbating muscle atrophy [3, 7]. Spaceflight and hindlimb suspension unloading have been found to cause atrophy and reloading damage to myofibrillar, including increasing susceptibility to interstitial edema and ischemic-anoxic necrosis, exhibiting muscle fiber tearing with disruption of contractile proteins and eccentric contraction-like lesions, among others [25, 26]. Given the high mitochondrial content of skeletal muscle, which is largely reliant on oxidative phosphorylation for energy production, ROS production primarily occurs within the mitochondria [27]. Increase in mitochondrial ROS production has been observed in the process of disuse-induced muscle atrophy and is considered a major trigger for imbalance between muscle protein synthesis and degradation under disuse conditions [28]. Furthermore, high levels of ROS have been demonstrated to result in mitochondrial dysfunction, inflammation, and/or autophagy [29, 30]. As expected, remobilization enhanced the production of ROS and the expression of total HIF-1α and ratio of nuclear HIF-1α to cytosolic HIF-1α in the rectus femoris within the first seven days of remobilization. The transcription factor HIF-1α regulates numerous hypoxic responses in cells and tissues. HIF-1α is subject to rapid degradation via the ubiquitin-proteasome system under conditions of normal oxygen levels and is stabilized by hypoxia. Previous research has shown that ROS cannot stabilize HIF-1α directly, but indirectly via hypoxia [31]. Other studies found that the mTORC1 signaling pathway plays a protective role in muscle atrophy and is activated during the acute recovery phase from hindlimb suspension [32, 33]. Notably, the mTORC1 pathway regulates HIF-1α levels under normoxic conditions, with HIF-1α levels increasing when the signaling pathway is activated and decreasing when inhibited by rapamycin. Additionally, previous research has shown that activation of mTORC1-HIF-1α pathway is associated with burn-induced muscle metabolic derangements and mitochondrial dysfunction [34]. Despite multiple studies have demonstrated that HIF-1α/BNIP3-dependent mitophagy can be triggered by ROS, the exact mechanisms underlying the upregulation of HIF-1α still require further investigation in future studies.

The accumulation of mitophagosomes within the first three days of remobilization observed by TEM provided the directly evidence for mitophagy activation. Based on previous research, mitophagy is also essential for maintaining muscle mass and healthy skeletal muscle, but overactivated mitophagy is not beneficial for the preservation of muscle mass. BNIP3, localized in the mitochondria and involved in autophagic clearance, is a marker for evaluating mitophagy [35]. In vivo inhibition of the mitophagy factor BNIP3 by RNAi has been shown to be sufficient to reduce muscle atrophy during fasting [14]. In this study, we examined the protein level of BNIP3 during remobilization and found significantly increased levels in group R-1d and group R-3d compared to group I-2w. The protein level of BNIP3 was significantly inhibited after one week, indicating that HIF-1α synchronously regulates BNIP3 to enhance activation of mitophagy in the rectus femoris at the early stage of remobilization. To support this claim, we assessed the protein level of mitochondrial protein and autophagy-associated protein. Our results showed that immobilization significantly reduced mitochondrial protein, such as HSP60 and COX IV in group I-2w compared to group C. Remobilization further reduced mitochondrial protein in group R-1d and group R-3d compared to group I-2w. At the early stage, immobilization significantly upregulated the expression of autophagy-associated protein, such as LC3B-II and Beclin-1 in group I-2w compared to group C, and remobilization further upregulated the expression of autophagy-associated protein. Importantly, remobilization further up-regulated the expression of autophagy-associated protein at the early stage. LC3B-II is the autophagosome-associating form of LC3B-I, and the conversion of LC3B-I to LC3B-II is necessary for autophagosome formation [36]. When autophagy is activated, cytoplasmic LC3B (LC3B-I) changes into membrane LC3B (LC3B-II). Beclin-1 is one of key autophagy related protein. Literatures reported that BNIP3 activates mitophagy by preventing the binding of Bcl-2 to Beclin-1, thereby freeing Beclin-1 to stimulate mitophagy [37]. Immunofluorescence staining of sections of rectus femoris provided further evidence for the presence of mitophagy in atrophic skeletal muscle at the early stage of remobilization. Considering the decreasing trend in total ATP content observed in the rectus femoris, we speculate that hyperactive mitophagy could lead to excessive elimination of healthy mitochondria, resulting in a shortage of ATP supply in the muscle recovering from atrophy. Hence, future research needs to confirm this. Based on our findings, we suggest that the inhibition of hyperactive mitophagy activation presents a potential therapeutic option for the worsening of skeletal muscle atrophy after immobilization.

Previous study indicated that BNIP3-mediated mitophagy involves the translocation of Parkin to mitochondria [38]. However, it can be seen that the protein level for PINK1 and Parkin remained stable in this present study. Previous research also showed that one week-immobilization or one week-remobilization of gastrocnemius did not change the protein levels of Parkin, but resulted in increased levels of BNIP3 mRNAs [16]. Furthermore, while immobilization induces both a reduction of muscle mass and fiber CSA in the tibialis anterior, the CSA of gastrocnemius fibers was stable during immobilization, albeit a decrease of muscle mass [16]. This phenomenon might be related with the fiber type proportions and the anatomical position during immobilization. In the present study, although the fiber type proportions of the rectus femoris is more similar to the gastrocnemius, we considered that immobilization/remobilization induced both a reduction of MWW/BW and CSA since the rectus femoris was fixed in a shortened position. Moreover, the mitochondrial biogenesis was also partially evaluated in this study, such as PGC-1α (a transcriptional co-activator promoting mitochondrial biogenesis). The results showed that mitochondrial protein was increased in group R-2w by compared with group 3d. We suspected that the myoblasts compensated for this adverse effect via up-regulating the expression of PGC-1α within the first seven days of remobilization [39]. Besides, it was reported that PGC-1α is a potent suppressor of ROS and induces the production of ROS-scavenging enzymes [40]. Previous research also revealed that intensified mitophagy in skeletal muscle with aging was downregulated by PGC-1α overexpression in rat [39]. Thus, up-regulating the expression of PGC-1α may be one of the factors ameliorating mitochondrial dysfunction during remobilization.

To our knowledge, this is the first study that demonstrated a hyperactive BNIP3-dependent mitophagy in skeletal muscle during remobilization, but this study also has certain limitations. Due to the limitation of experimental conditions and selection of study subject, this study did not use gene knockout or specific mitophagic inhibitor to directly study the effect of skeletal muscle mitophagy. We will further test the present findings through in vitro experiment in our future studies. Furthermore, we acknowledge that the detection of ATP content might be best performed using fresh skeletal muscle tissue, but the preservation method employed in our study did not satisfy these conditions. In addition, we did not study whether ubiquitin-proteasome-dependent proteolysis pathway, a closely related to muscle atrophy, is related to mitophagy. Therefore, we hope that future studies will address these limitations and provide further insights into the worsening of skeletal muscle atrophy during remobilization.

## Conclusions

In conclusion, this study suggested that BNIP3-denpendent mitophagy was sustained activated at the early stages of remobilization, and it might be one of the causes of the worsening of skeletal muscle atrophy induced by immobilization. These results might provide a theoretical basis for the worsening of skeletal muscle atrophy during remobilization and provide a potential therapeutic target.

### Electronic supplementary material

Below is the link to the electronic supplementary material.

Supplementary Material 1

Supplementary Material 2

## Data Availability

The datasets used and analyzed during the current study are available from the first author on reasonable request. All data generated or analyzed during this study are included in this published article. The manuscript, including related data, figures and tables have not been previously published and are not under consideration elsewhere.

## Abbreviations

HIF-1α: Hypoxia-inducible factor-1α
LC3: Microtubule-associated protein 1 light chain 3
ROS: Reactive oxygen species
CSA: Cross-sectional area
MWW/BW: Muscle wet weight/body weight
DHE: Dihydroethidium
