# Supplementary material for: The worsening of skeletal muscle atrophy induced by immobilization at the early stage of remobilization correlates with BNIP3-dependent mitophagy
Source: BMC Musculoskelet Disord. 2023 Aug 4;24:632. doi: 10.1186/s12891-023-06759-2 (PMC10401904; doi:10.1186/s12891-023-06759-2)

## Figure 4. C Total HIF-1α

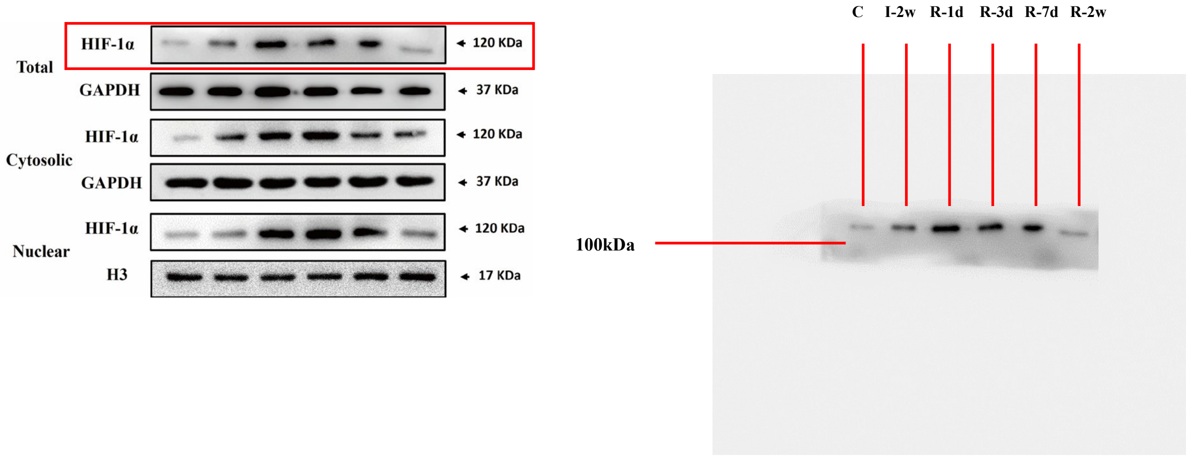


## Total HIF-1α (Repeated)

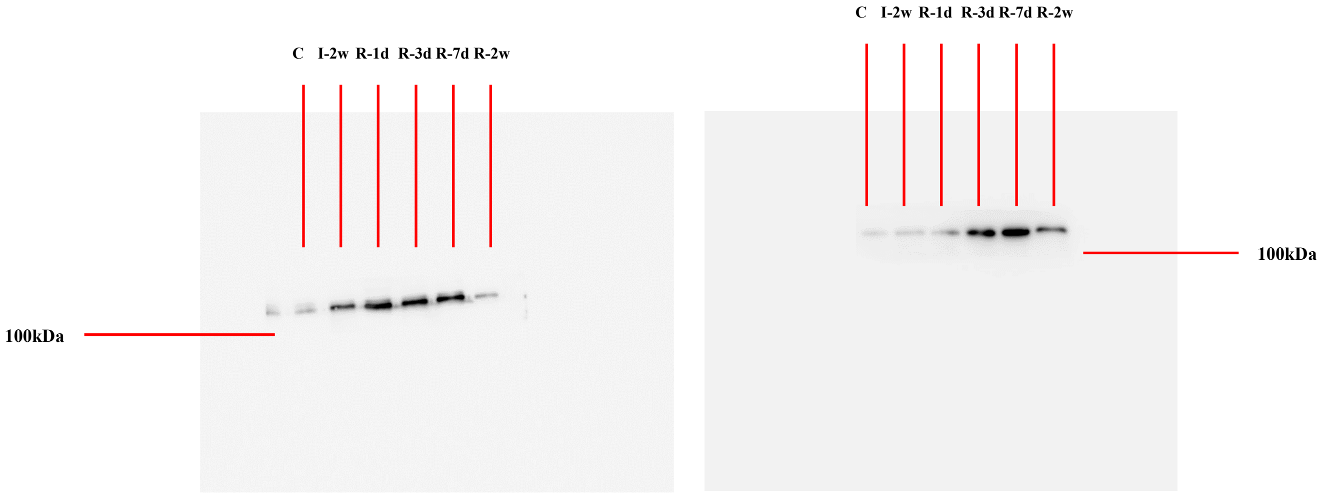


## Figure 4. C Total GAPDH

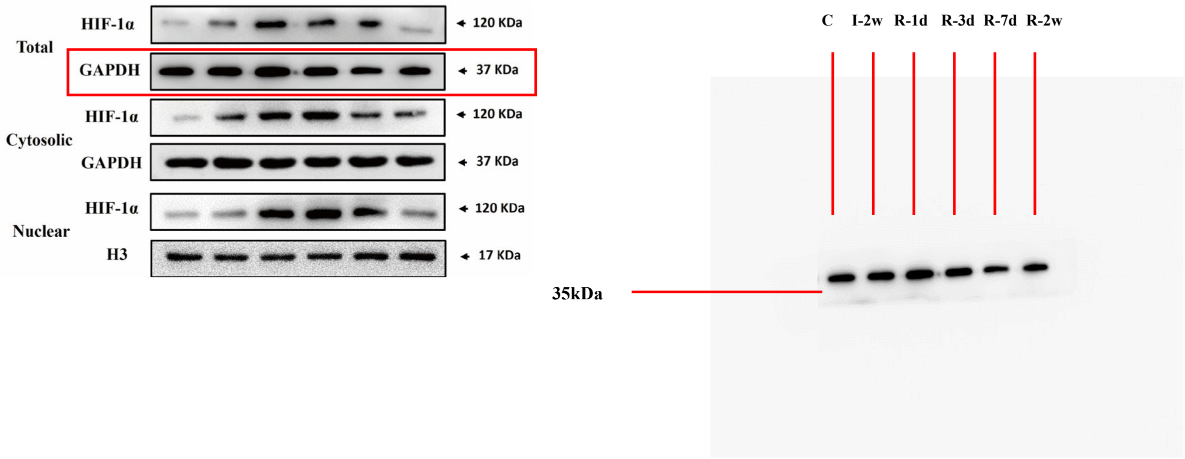


## Total GAPDH (Repeated)

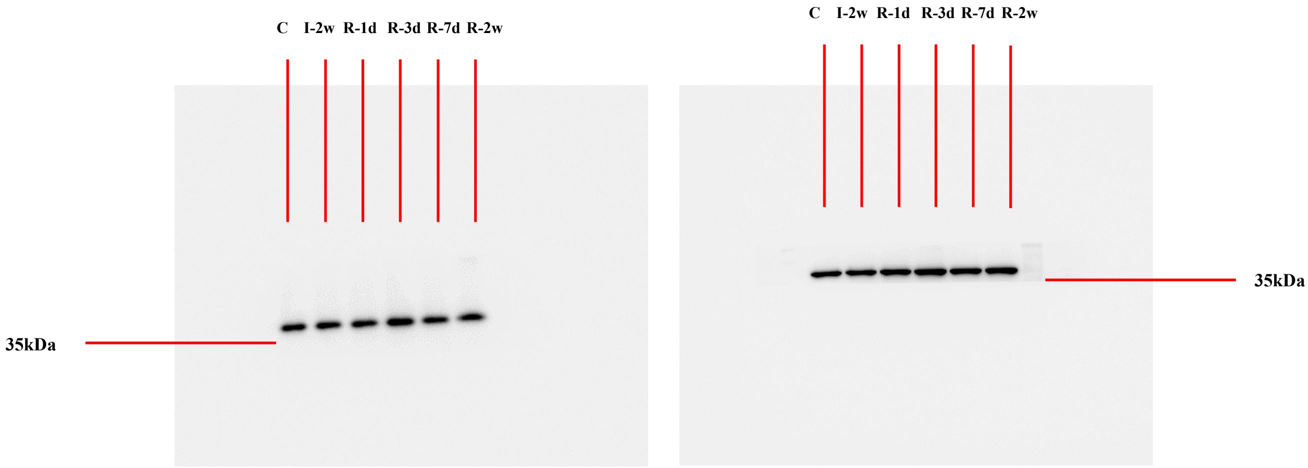

## Figure 4. C Cytosolic HIF-1α

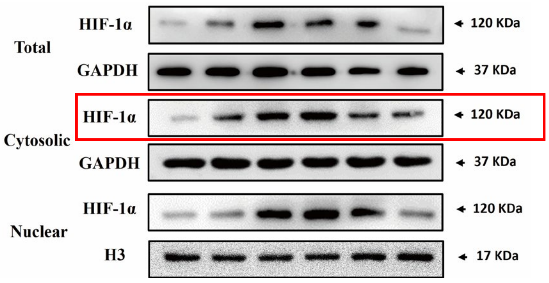


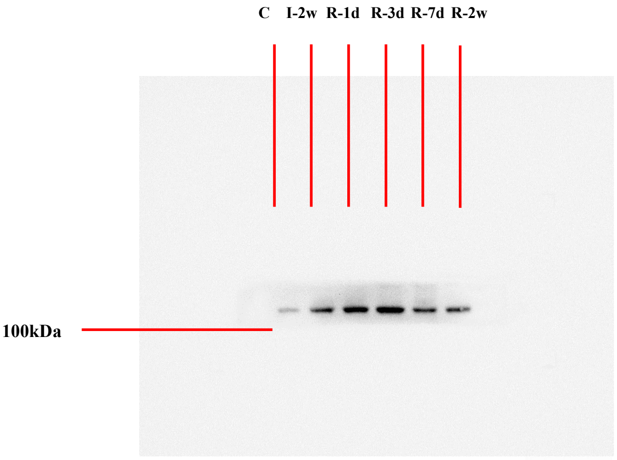


## Cytosolic HIF-1α (Repeated)

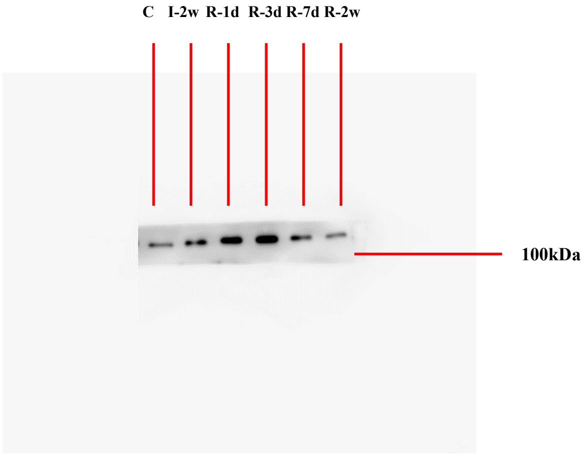


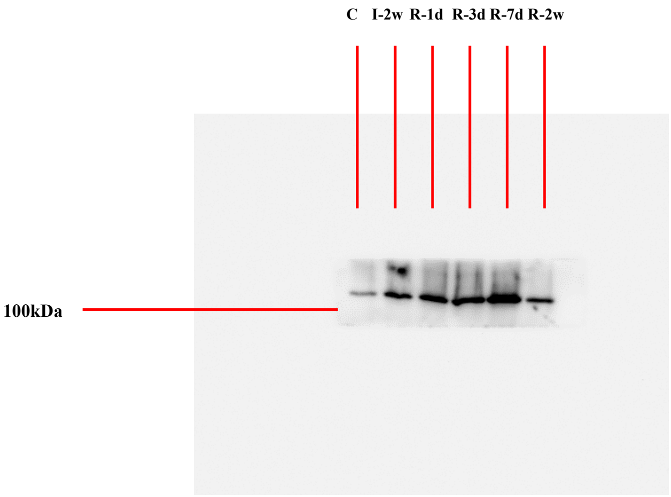


## Figure 4. C Cytosolic GAPDH

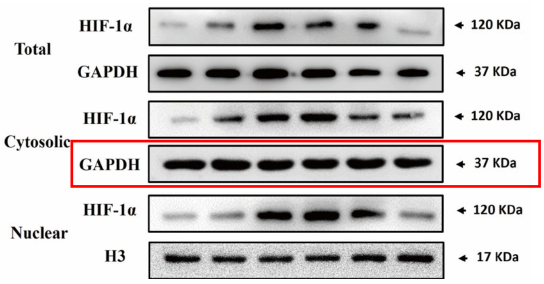


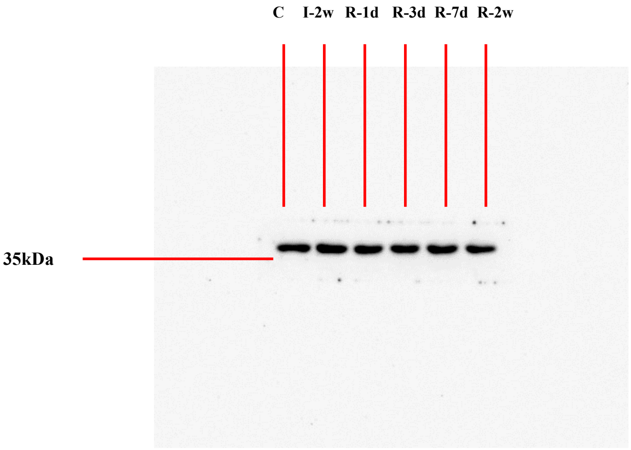


## Cytosolic GAPDH (Repeated)

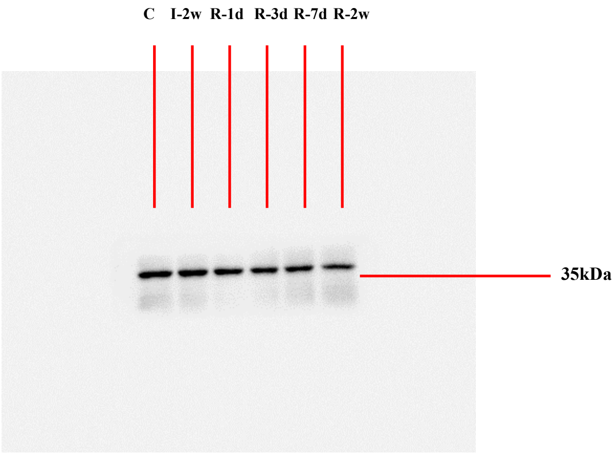


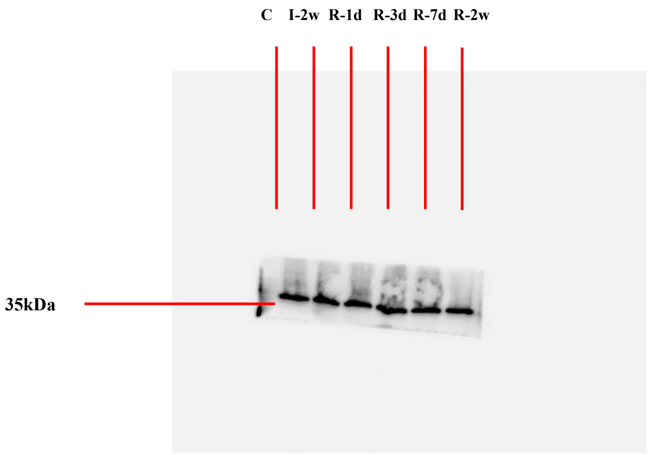

## Figure 4. C Nuclear HIF-1α

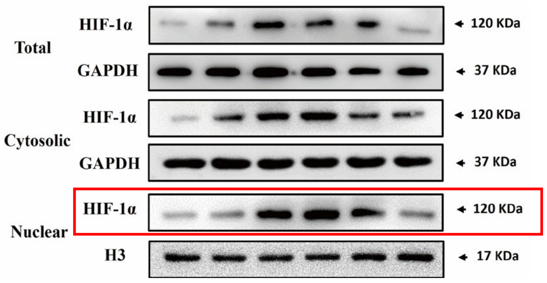


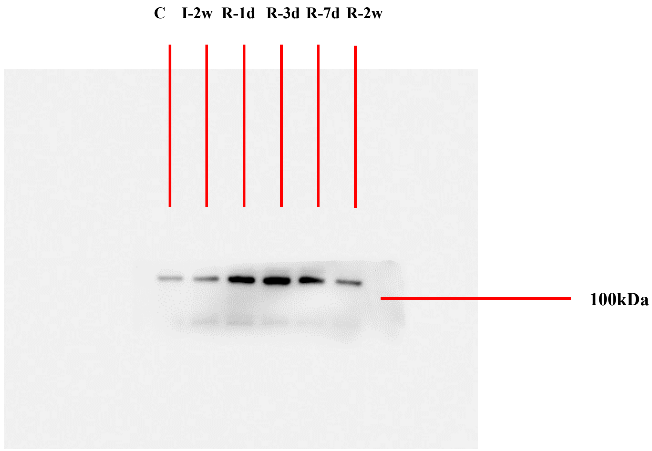


## Nuclear HIF-1α (Repeated)

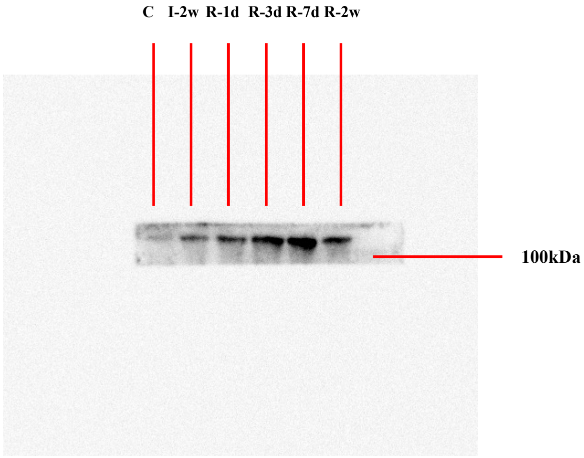


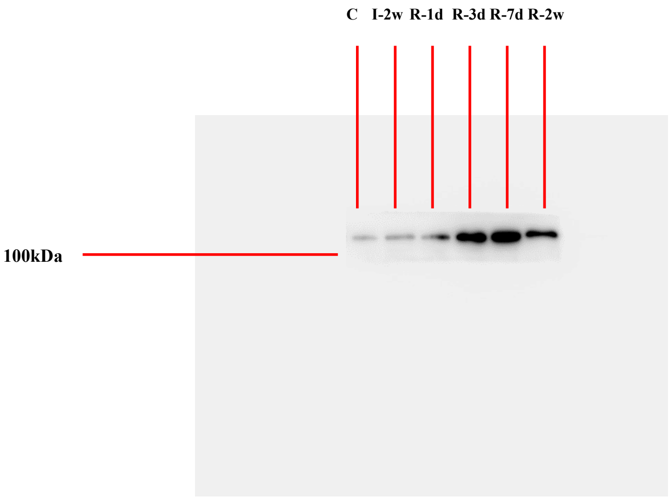


## Figure 4. C Nuclear H3

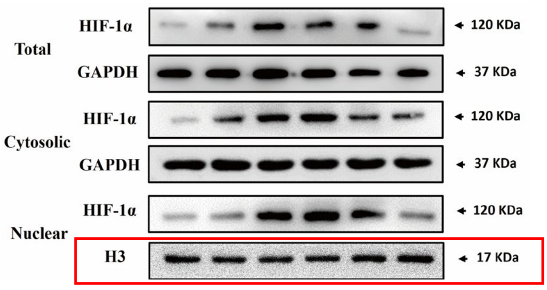


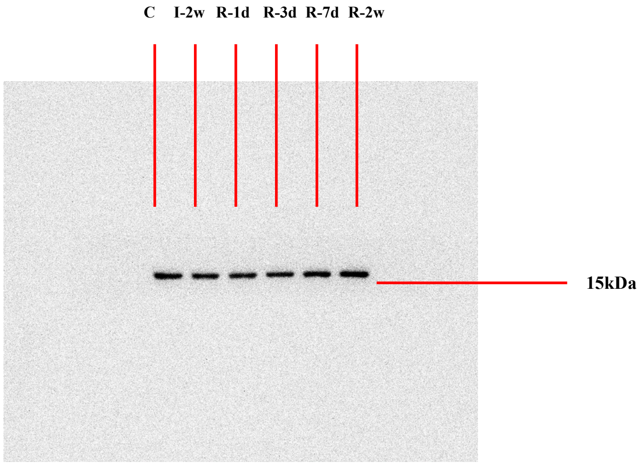


## Nuclear H3 (Repeated)

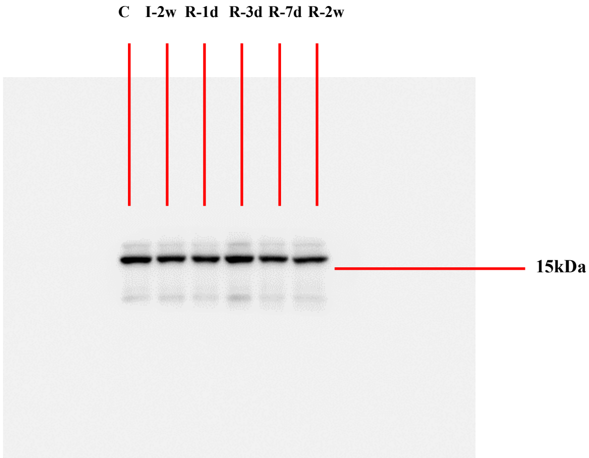


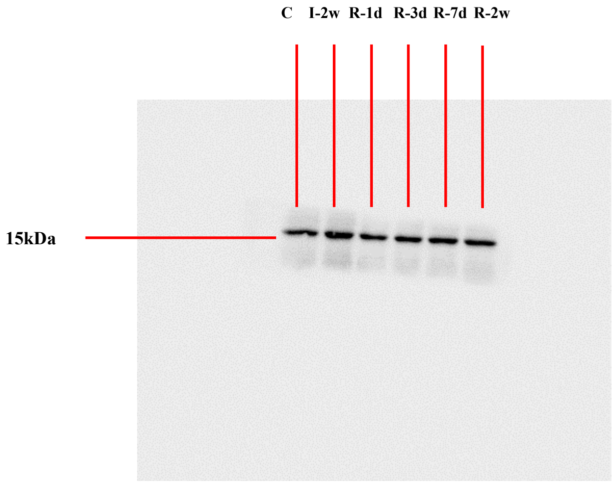

## Figure 5. B PGC-1α

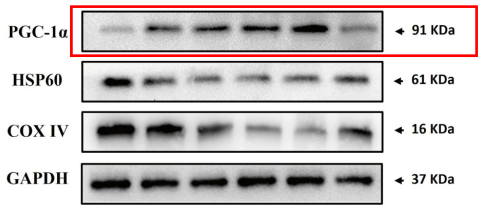


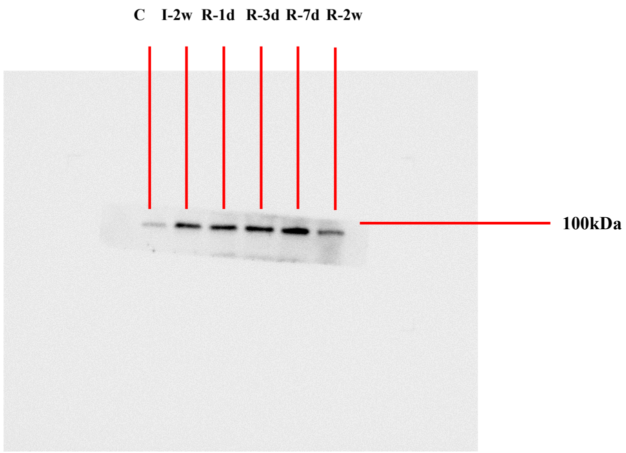


## PGC-1α (Repeated)

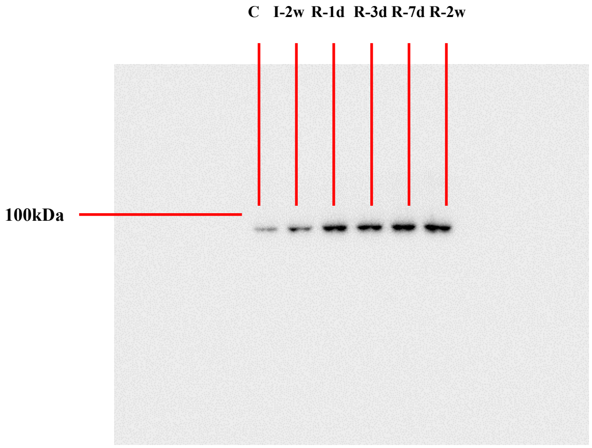


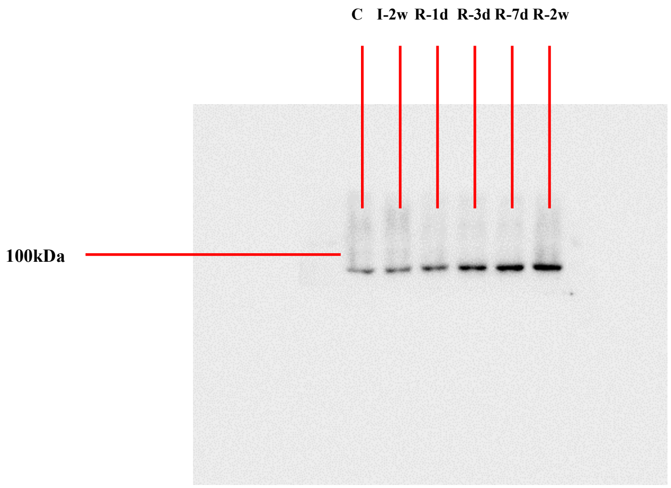


## Figure 5. B HSP60

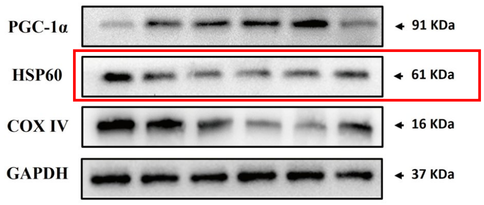


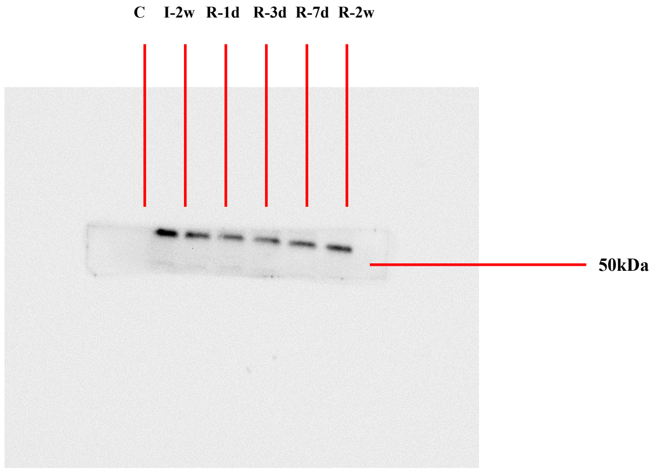


## HSP60 (Repeated)

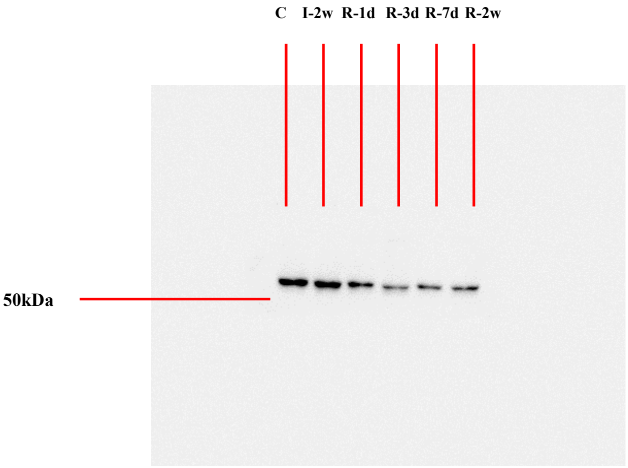


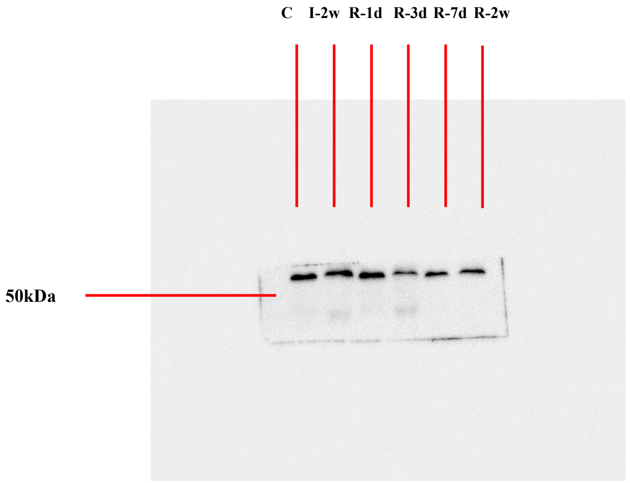

## Figure 5. B COX IV

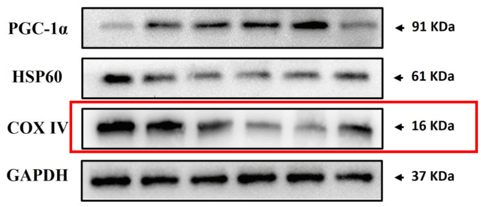


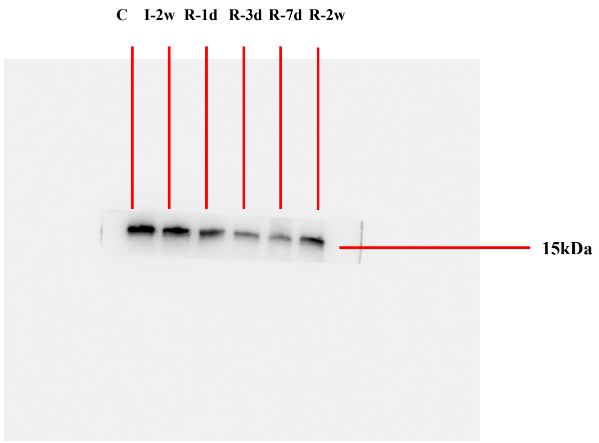


## COX IV (Repeated)

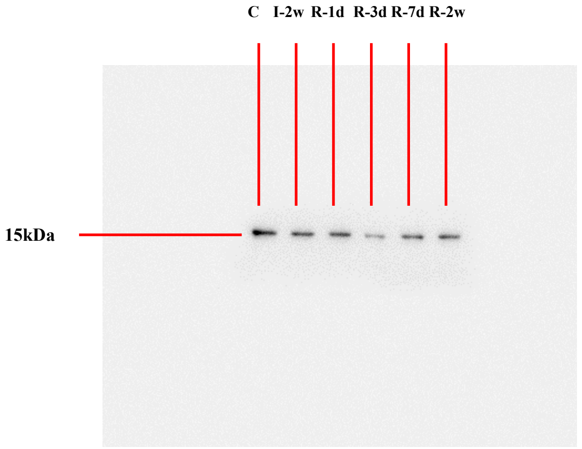


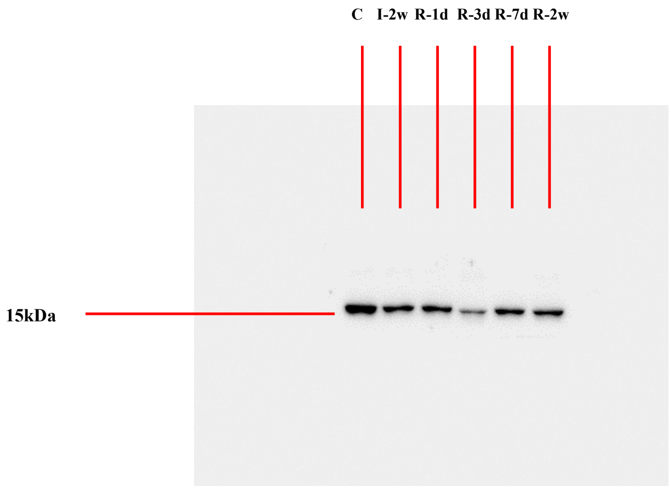


## Figure 5. B GAPDH

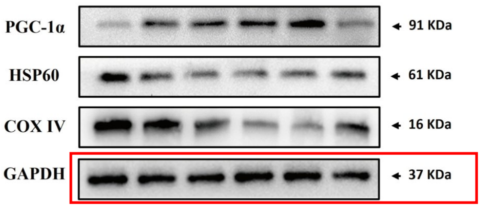


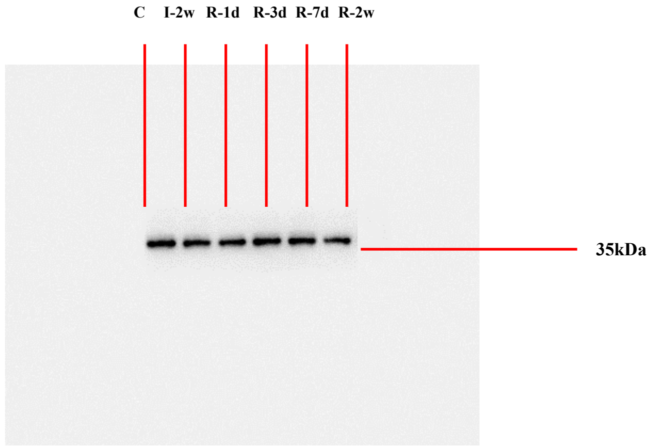


## GAPDH (Repeated)

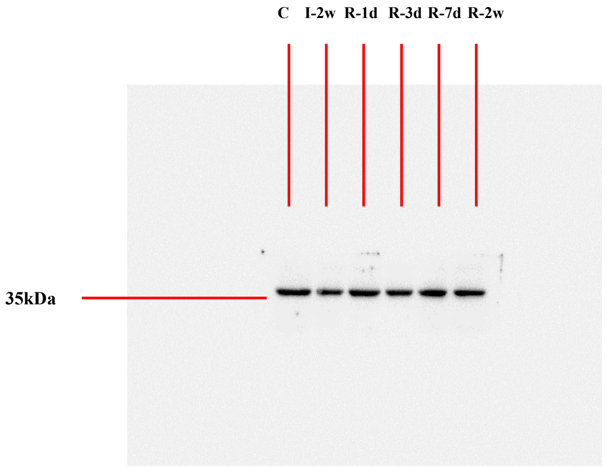


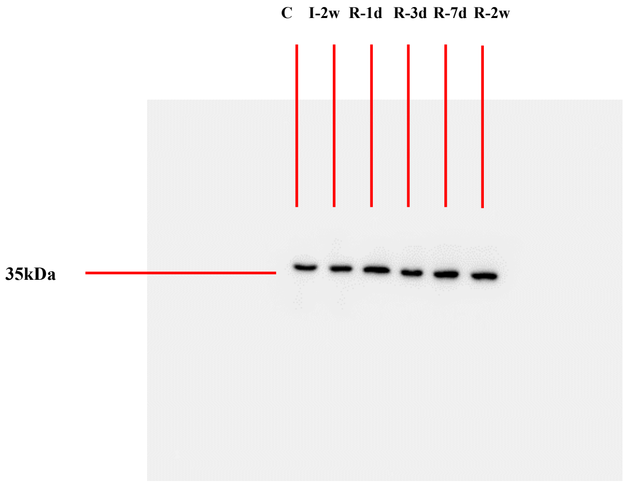

## Figure 5. C PINK1

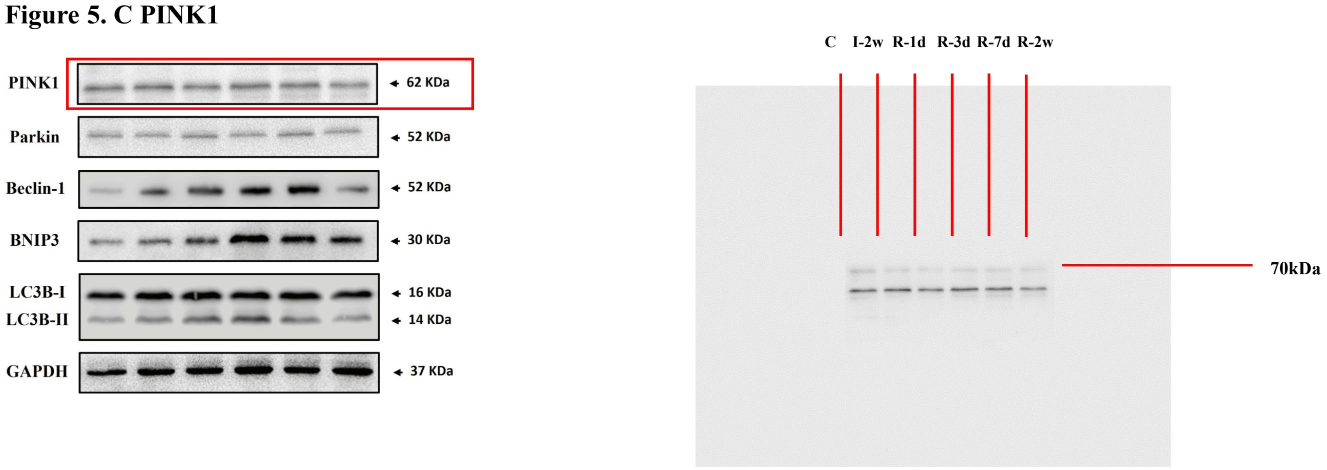


## PINK1 (Repeated)

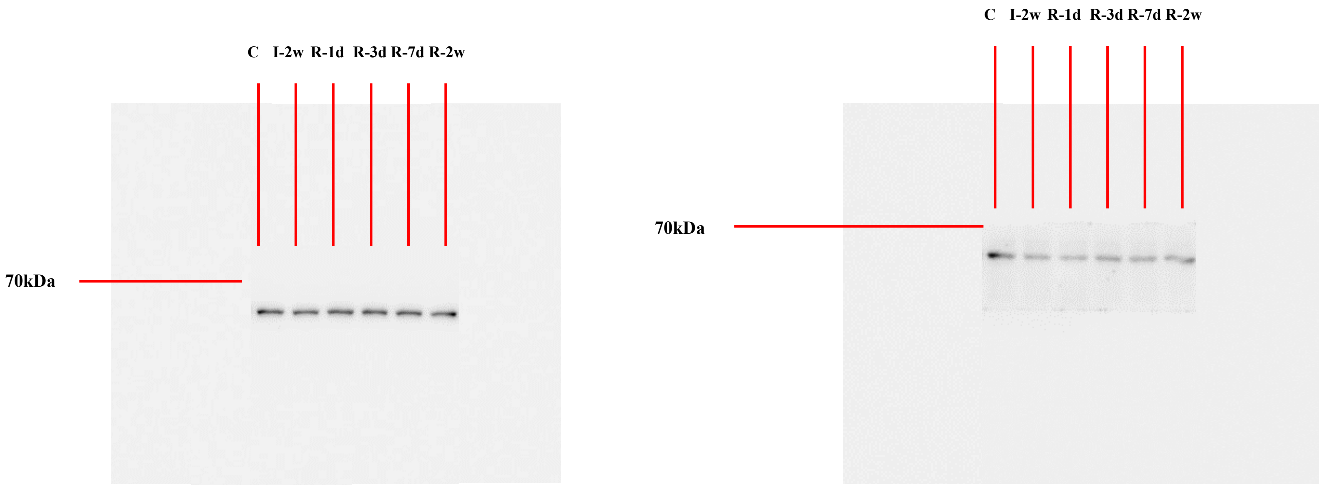


## Figure 5. C Parkin

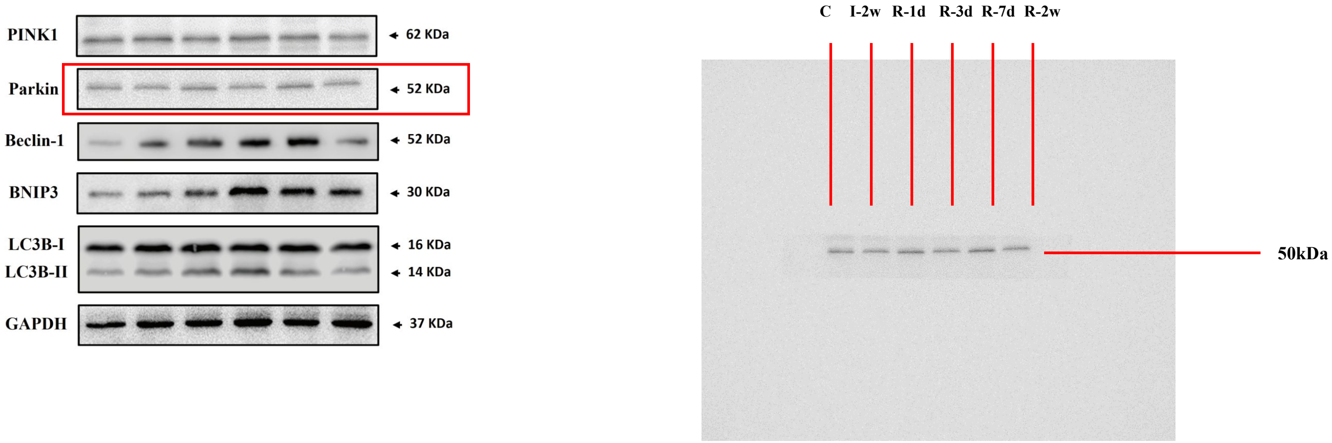


## Parkin (Repeated)

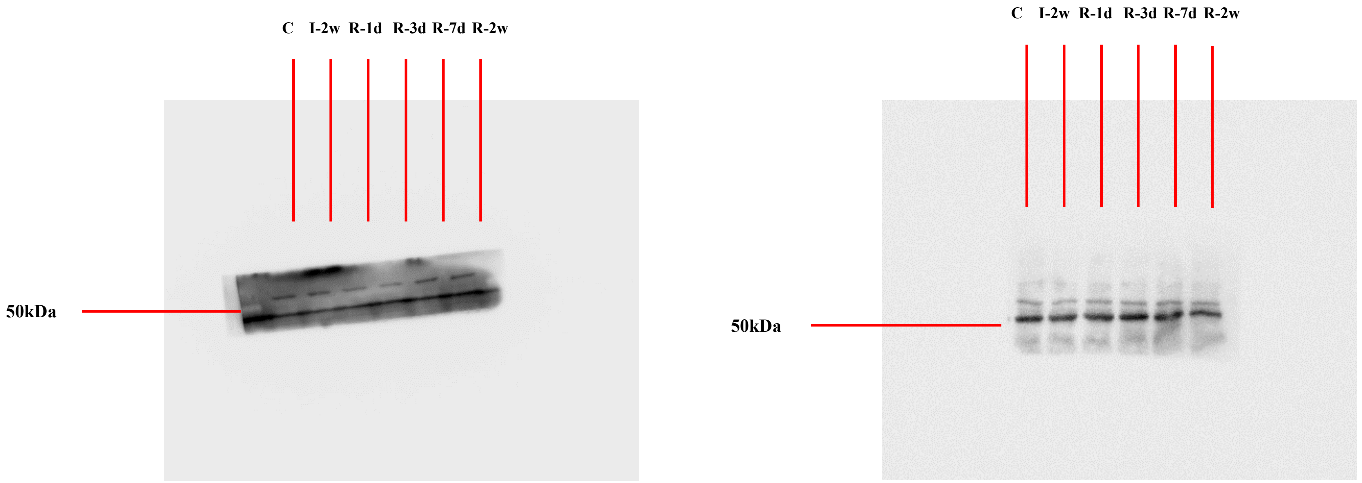

## Figure 5. C Beclin-1

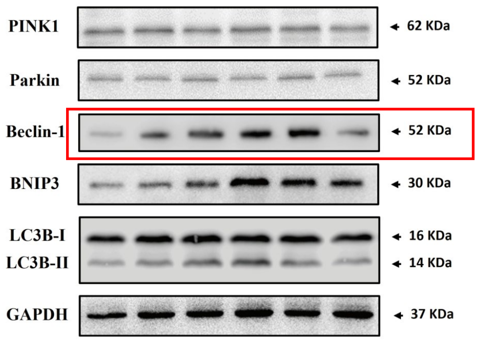


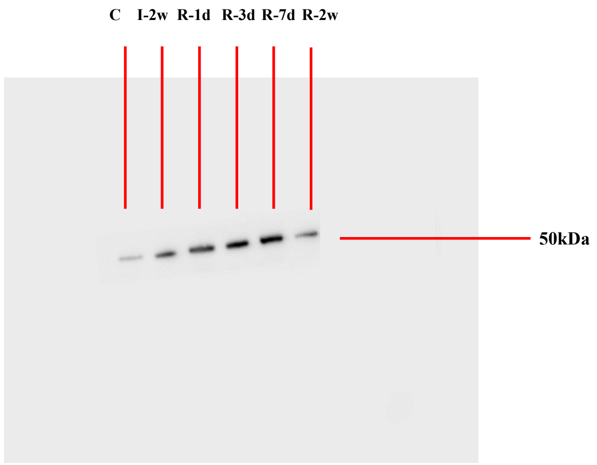


## Beclin-1 (Repeated)

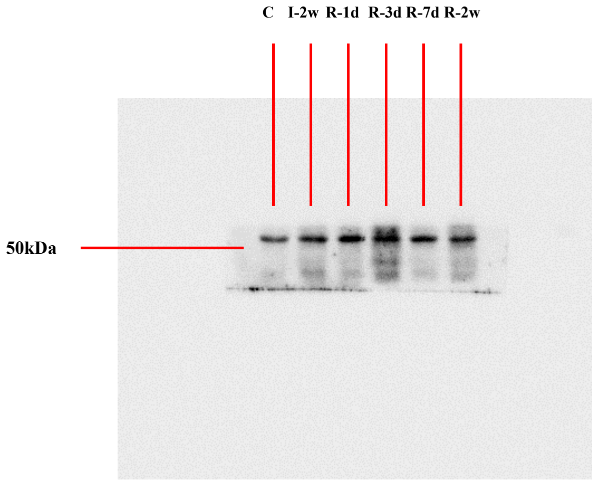


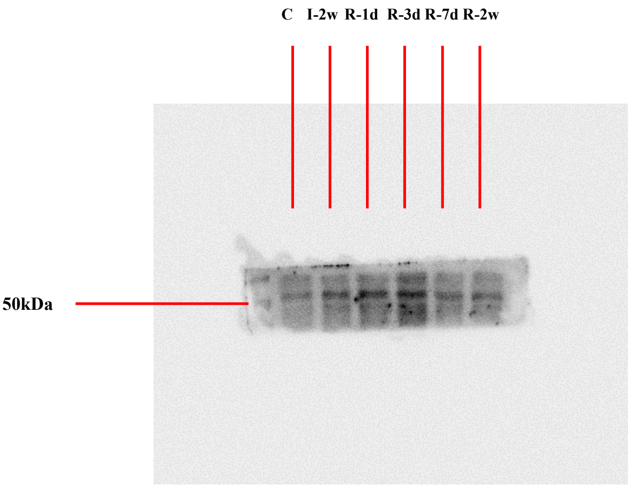


## Figure 5. C BNIP3

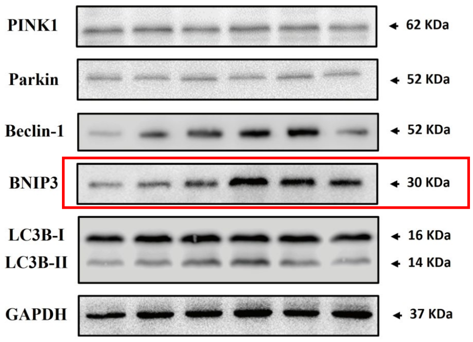


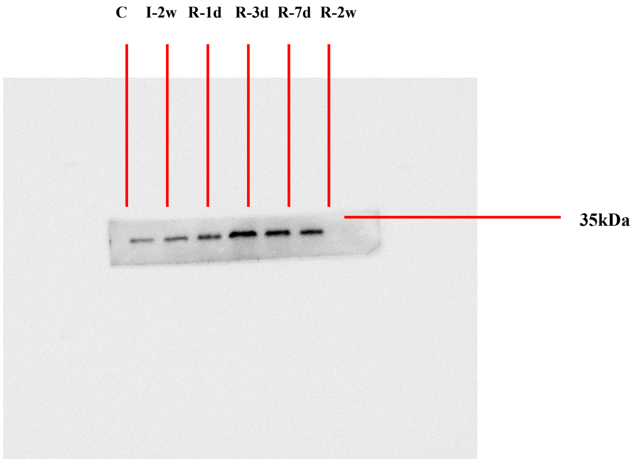


## BNIP3 (Repeated)

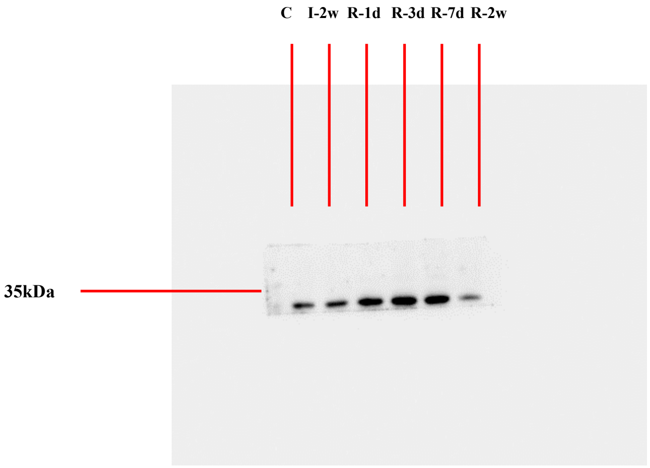


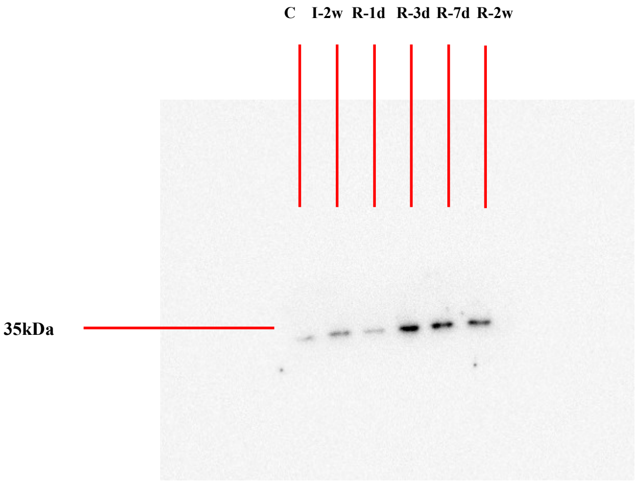

## Figure 5. C LC3B

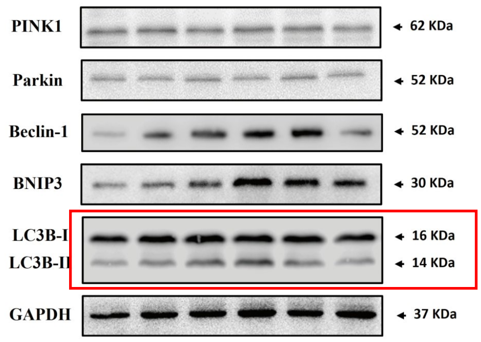


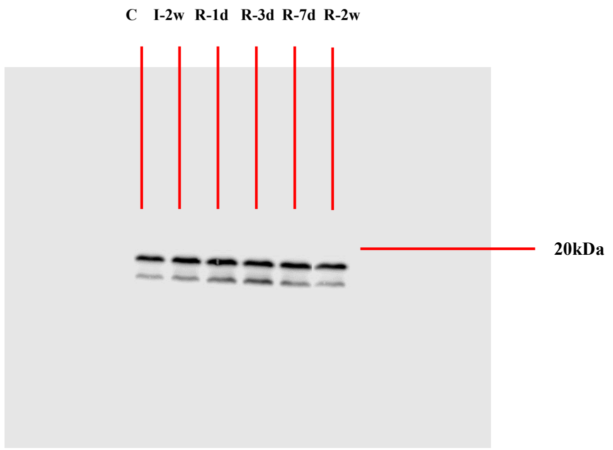


## LC3B (Repeated)

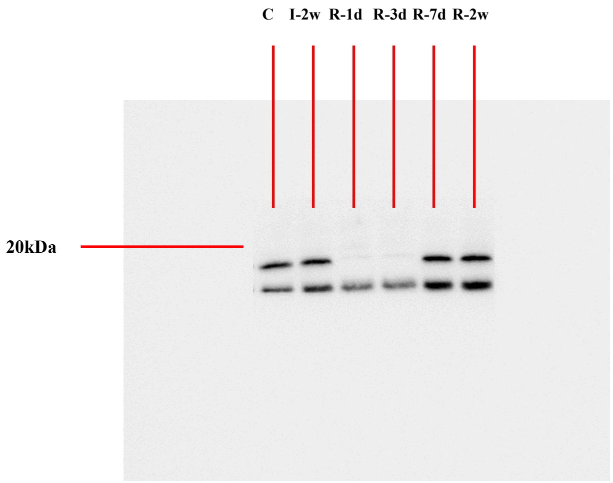


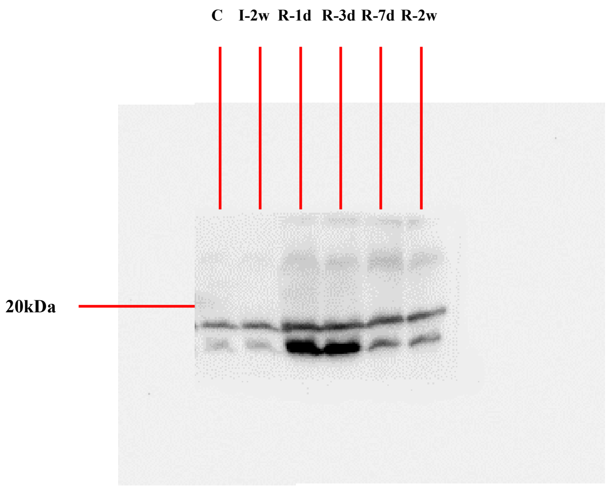


## Figure 5. C GAPDH

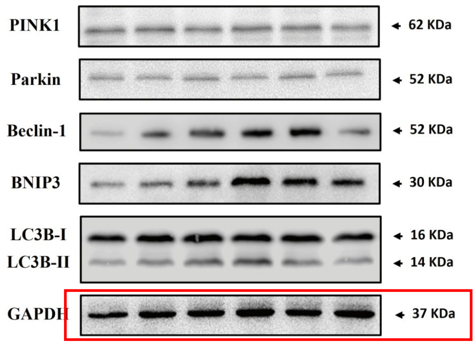


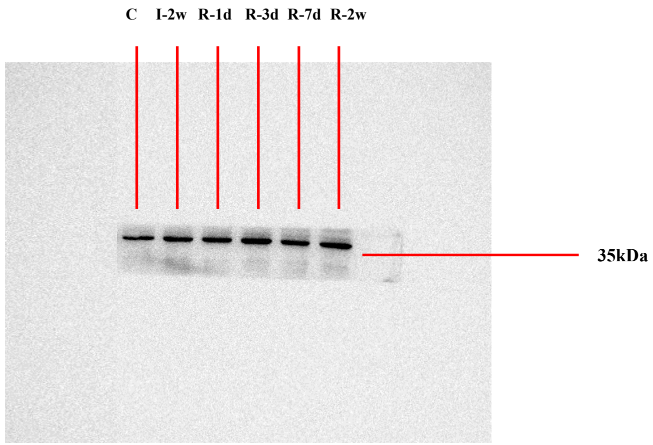


## GAPDH (Repeated)

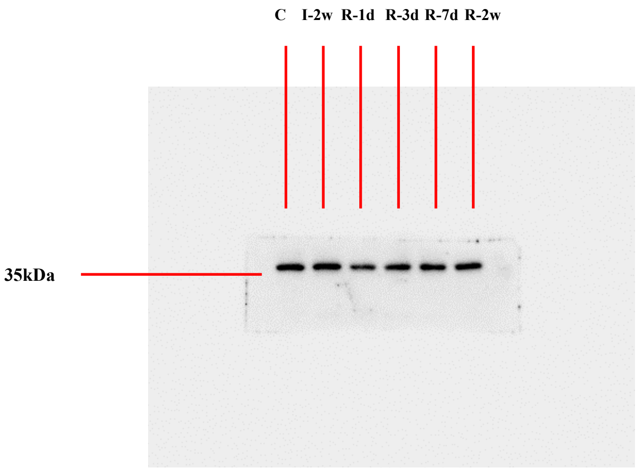


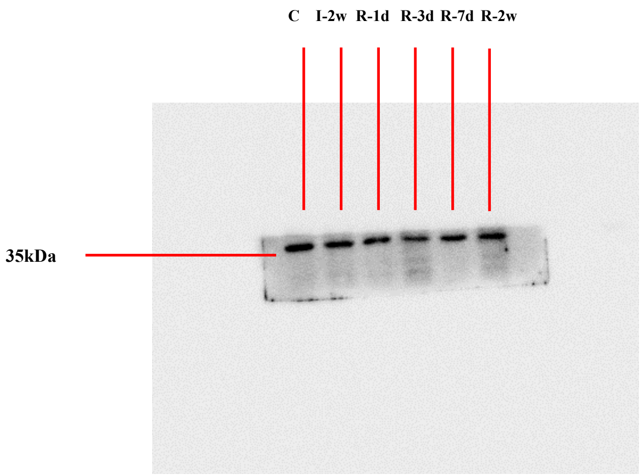

Supplement: Supplementary file 1 — Supplementary Material 1 [file 12891_2023_6759_MOESM1_ESM.pdf]
